# Identification of Novel Human Damage Response Proteins Targeted through Yeast Orthology

**DOI:** 10.1371/journal.pone.0037368

**Published:** 2012-05-16

**Authors:** J. Peter Svensson, Rebecca C. Fry, Emma Wang, Luis A. Somoza, Leona D. Samson

**Affiliations:** 1 Biological Engineering Department, Center for Environmental Health Sciences, Massachusetts Institute of Technology, Cambridge, Massachusetts, United States of America; 2 Computation and Systems Biology, Massachusetts Institute of Technology, Cambridge, Massachusetts, United States of America; 3 Department of Biology, Massachusetts Institute of Technology, Cambridge, Massachusetts, United States of America; Institute of Enzymology of the Hungarian Academy of Science, Hungary

## Abstract

Studies in *Saccharomyces cerevisiae* show that many proteins influence cellular survival upon exposure to DNA damaging agents. We hypothesized that human orthologs of these *S. cerevisiae* proteins would also be required for cellular survival after treatment with DNA damaging agents. For this purpose, human homologs of *S. cerevisiae* proteins were identified and mapped onto the human protein-protein interaction network. The resulting human network was highly modular and a series of selection rules were implemented to identify 45 candidates for human toxicity-modulating proteins. The corresponding transcripts were targeted by RNA interference in human cells. The cell lines with depleted target expression were challenged with three DNA damaging agents: the alkylating agents MMS and 4-NQO, and the oxidizing agent t-BuOOH. A comparison of the survival revealed that the majority (74%) of proteins conferred either sensitivity or resistance. The identified human toxicity-modulating proteins represent a variety of biological functions: autophagy, chromatin modifications, RNA and protein metabolism, and telomere maintenance. Further studies revealed that MMS-induced autophagy increase the survival of cells treated with DNA damaging agents. In summary, we show that damage recovery proteins in humans can be identified through homology to *S. cerevisiae* and that many of the same pathways are represented among the toxicity modulators.

## Introduction

Sensing, signaling and repair of DNA damage requires many proteins [1] and depletion of any one of these proteins may affect cellular survival after DNA damage. DNA damaging agents, from both endogenous and exogenous sources, constantly challenge genome integrity, causing mutations, permanent cell cycle arrest and cell death. The two latter endpoints can be exploited for therapeutic purposes. For example, a common class of cancer chemotherapy agents are DNA damaging agents that act by alkylation, as represented by the drugs Temozolomide and Carmustine (1,3-bis(2-chloroethyl)-1-nitrosourea, BCNU) [2], [3]. Other alkylating agents include the extensively studied model agents methyl methanesulfonate (MMS) and 4-nitroquinoline-N-oxide (4-NQO) that have been used to explore the DNA damage responses of cells and organisms (reviewed in [4]). The simple S_N_2 alkylating agent MMS attacks DNA, forming products that include 7-methylguanine and the highly toxic 3-methyladenine [5]. These lesions can be efficiently removed by DNA glycosylases like AAG/MPG in mammals to initiate the base excision pathway [6]. Damage induced by the bulky alkylating agent 4-NQO requires a more complex arsenal of repair capacities [7], [8], [9]. The large DNA base adducts formed by the metabolically activated 4-NQO stall both transcription and replication, as does 3-methyladenine (3MeA), but in contrast, 4-NQO induced lesions are not necessarily as toxic as 3MeA [10]. Many of the 4-NQO induced lesions require nucleotide excision repair to be resolved [9]. Also, in the process of activation, 4-NQO metabolism generates reactive oxygen species, causing oxidative damage to cellular components. Another pro-oxidant is the oxidizing agent tert-butyl hydroperoxide (t-BuOOH), which has many effects on cell metabolism [11].

All of the mentioned DNA damaging agents have been shown to modulate the expression of many genes, and cells lacking a wide variety of proteins show aberrant responses to DNA damage [12], [13], [14], [15], [16], [17], [18], [19], [20], [21], [22], [23], [24], [25]. Indeed, recent genome-wide siRNA screens in human cells have revealed many unexpected pathways involved in maintaining genome stability [19], [22], [23]. In budding yeast, extensive studies of deletion mutants have revealed that approximately 30% of the genes affect recovery after damage with alkylating agents. Previous studies from our group determined yeast survival in libraries of gene deletion mutants after exposure to four DNA damaging agents (MMS, 4-NQO, t-BuOOH and UV). Distinct toxicity profiles were identified for each agent, and surprisingly, very few gene deletion strains were sensitive to all four agents. Similar screens of toxicity-modulating proteins have been conducted for MMS in *Drosophila* [26] and for ionizing radiation in *C. elegans* [27], showing comparable results. The toxicity-modulating proteins represent a variety of biological functions and biochemical pathways. Apart from proteins involved in stress signaling, cell cycle control, DNA repair and cell death, functions such as transcription, vesicle transport, protein and RNA metabolism, and telomere maintenance also affect recovery after exposure to DNA damaging agents. However, the direct role of these processes in damage recovery remains largely unknown.

In this study, we aimed to identify novel pathways needed for human cells to recover from exposure to DNA damaging agents. We hypothesized that the yeast data combined with the human protein interactome could be used to pinpoint human proteins needed for recovery, thus identifying novel damage response pathways in humans. Based on the results from *S. cerevisiae* [13], we used computational techniques to identify human protein candidates of toxicity modulation. 45 human proteins, spanning the human pathways identified as toxicity-modulating in *S. cerevisiae*, were tested for their role in the recovery of human cells after damage. We found that 74% of the proteins tested modulated the survival of human cells.

## Results

### Identification of human homologs of toxicity-modulating yeast proteins

**Figure 1:**
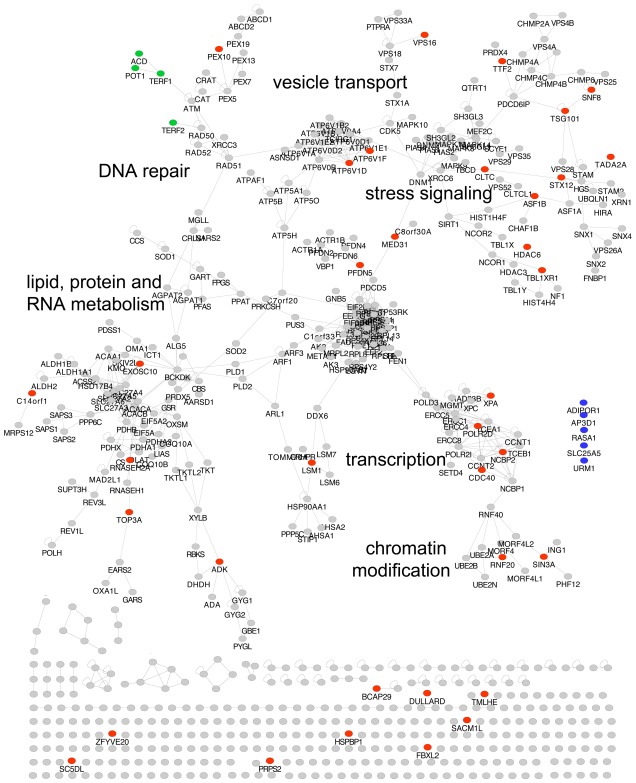
Human interaction network shows high connectivity among putative human toxicity-modulating proteins homologous to toxicity-modulating proteins in yeast. The largest connected component of the human interactome selected from yeast orthologs being required for damage recovery after treatment with MMS, 4NQO, t-BuOOH and UV [13]. The circles represent: red – proteins with toxicity-modulating yeast homologs targeted for silencing in this study; grey – proteins with toxicity-modulating yeast homologs not targeted in this study; blue – proteins with non-toxicity-modulating yeast homologs targeted in this study; green –proteins specific for mammalian telomere maintenance targeted in this study. An interactive version of this figure is available at http://www.bionut.ki.se/users/pesv/MIT/fig1.html.

The aim of this study was to determine whether the human homologs of toxicity-modulating proteins in yeast, spanning a wide range of cellular functions, also play roles in the damage response of human cells. First, human-yeast protein homologs were identified based on amino acid sequence similarity. Toxicity-modulating proteins were selected from [13]. Two public databases, Ensembl and Inparanoid, were used to identify 1,368 homologs of the 4,733 proteins represented in the *S. cerevisiae* gene deletion library. Of these, 646 human proteins were identified as homologous to yeast proteins with toxicity-modulating properties for at least one of the four DNA damaging agents used in the yeast study. The homologs were projected onto a human protein-protein interaction network previously described [28]. Surprisingly, 44% of the nodes, representing 284 proteins, were connected in one large connected component (p<1×10^−16^, permutation test), indicating that although the proteins are involved in disparate functions, a large proportion of them are connected by protein-protein interactions (Figure 1, an interactive version at http://www.bionut.ki.se/users/pesv/MIT/fig1.html). In the large connected component of the interactome, numerous biological categories are represented, including DNA repair, stress signaling, vesicle transport, chromatin modification, plus lipid, protein and RNA metabolism. The network is highly modular and most of the functional categories represented in yeast are also represented in the human network, with the exception of telomere maintenance (Tables S1a and S1b). Telomere stability is maintained by non-homologous proteins in *S. cerevisiae* and mammals and therefore a group of telomere-specific human proteins were queried separately.

### Selection of putative toxicity-modulating human proteins

To reduce the number of targets from all the human homologs of toxicity-modulating yeast proteins, a set of selection rules was implemented. In the previous study of yeast proteins in a library of deletion strains [13], only 28 strains were sensitive to all four of the tested DNA damaging agents. Twelve of the 28 proteins had human homologs and were included in this screen; these proteins display heterogeneity in cellular functions (Table S2). Additional targets were selected from the large interconnected sub-network. Proteins with already established roles in DNA repair or cell cycle control were excluded, as were ribosomal proteins. Preference was given to proteins with only one human homolog to a specific yeast protein, and to proteins with several protein-protein interactions. In addition, genes had to be expressed at reasonable levels in human cells as measured in a previous study [29]. We also gave preference to proteins that were among the highly represented categories in yeast, such as transcription, chromatin remodeling, vesicle transport and protein/mRNA degradation. In light of the involvement of telomere maintenance among the toxicity-modulating yeast proteins, four proteins in the shelterin complex were also selected since the shelterin complex is specific for telomere maintenance in mammalian cells. A brief summary of the 45 selected proteins, including a description of functions, GO terms and yeast homologs and their sensitivity, can be found in Table S2.

### Efficient reduction of mRNA levels in 293T cells

**Figure 2:**
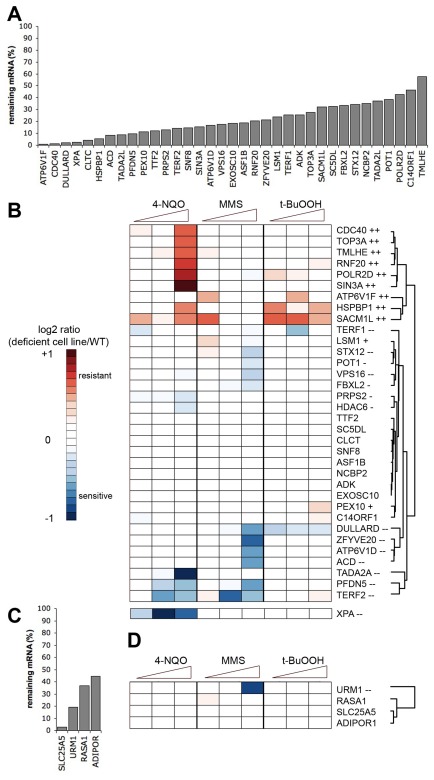
The majority of the selected proteins modulate the recovery after damage from the three compounds MMS, 4-NQO and t-BuOOH. A) RNA levels of shRNA targeted genes in 293T cells were measured by qRT-PCR and compared to cells infected with non-silencing control shRNA. B) Survival of cells depleted of target proteins exposed to three DNA damaging agents as revealed by heatmap. The color represents sensitivity to the damaging agent compared to the cell lines with non-silenced targets. ++ indicate high resistance. + low resistance, − high sensitivity, − low sensitivity. C) Knock-down of human homologs of non-toxicity modulating proteins in yeast, as measured by qRT-PCR. D) Survival of cells depleted of human homologs of non-toxicity modulating proteins in yeast. Colors and symbols are the same as in B.

RNA interference was used to deplete the transcript levels of the selected targets in human cells. Stable clonal cell lines were created after lentiviral infection of shRNAs targeting the mRNA of selected genes. We used the adherent embryonic kidney cell lines 293T as the parental cell line since these cells readily and stably express foreign DNA. For 35 gene targets we achieved a reasonable knock-down effect (<60% residual mRNA level compared to controls) in the 293T background (Figure 2A).

### The large majority of selected homologs are toxicity-modulating in human cells

To test whether deficiency for the targeted proteins resulted in altered sensitivity to DNA damaging agents, the cells with reduced levels of the target mRNA were exposed to three different damaging agents at equitoxic doses: the alkylating agents MMS and 4-NQO and the oxidizing agent t-BuOOH. The cell lines were always compared to control experiments performed contemporaneously. The variation between days was minimal, as determined by the repeated survival data of the control cell line expressing an shRNA construct targeting a sequence not present in the human genome (data not shown). To account for off-target effects, four non-silenced cell lines expressing an shRNA construct were tested for survival after treatment with the damaging agents. These non-silenced cell lines expressed target shRNA against RNASEH2A, TBL1XR1, AP3D1 and a non-silencing clone of ATP6V1F had no significant effect on the target gene expression (>60% residual levels of target RNA, Figure S1A). The sensitivity range of these cell lines together with the range of cells expressing shRNA targeting a sequence not present in the human genome were set as the detection limits of this screen (Figure S1B–C, grey and black lines). The survival data of the cell lines with confirmed targeted gene silencing is summarized in a heatmap (Figure 2B). XPA-deficient cells were included as a positive control. XPA is a DNA repair protein know to be important for the repair of UV-induced lesions [30]. Here we show that lack of XPA lead to a specific reduction in survival after treatment with the UV-mimetic 4-NQO. In summary, for 34 targets that were not previously associated with the DNA damage response, we obtained significant and reproducible results regarding their effect on sensitivity to three DNA damaging agents. These data show that reduced transcript levels of 14 of the 34 proteins (41%) conferred high (>25% different from WT) or moderate (20–25% different from WT) sensitivity to DNA damaging agents in cells. Surprisingly, as target proteins were selected based on the sensitivity of yeast deletion mutants, 11 (32%) of the human cell lines showed high or moderate resistance to DNA damaging agents. In total, 19 of the 34 proteins (56%) showed high (>25% different from WT) toxicity-modulation (see Material and Methods section for details). Six additional proteins showed moderate (20–25% different from WT) toxicity-modulation (FBXL2, POT1, PEX10, HDAC6, PRPS2, and LSM1), bringing the total percentage of toxicity-modulating proteins in our selection to 74%.

### A random selection of human proteins contains a low proportion of toxicity-modulating proteins

Given that 74% of the targeted proteins caused a toxicity-modulating phenotype, we then sought to estimate what would be found by random chance. We hypothesized that human deficiency of homologs of yeast proteins that did not modulate toxicity in yeast would likewise not result in sensitivity changes to the DNA damaging agents in human cells. To test this hypothesis, we identified the proteins with no evidence of toxicity-modulation in yeast [13]. Out of the 724 yeast-human homologs of non-toxicity-modulators, 200 genes were expressed in human cell lines [29]. Five of these proteins were selected completely at random: SLC25A5, AP3D1, ADIPOR1, URM1, RASA1. For four proteins, reduced mRNA levels (<60%) were achieved. For cell lines lacking these proteins, the survival after treatment with the DNA damaging agents was determined. A deficiency for only one of the four proteins, URM1, resulted in an altered sensitivity phenotype. URM1 was recently described to affect cellular recovery after starvation and oxidative stress [31]. Despite the small number of cell lines tested here, we conclude that 74% of toxicity-modulating proteins in our screen appears to be different from the random sampling of proteins (borderline significance, p = 0.08 (Fischer's exact test)). Therefore, the selection of yeast-human homologs seems advantageous in discovering new mammalian toxicity-modulating proteins, although we were not able to predict the direction of the toxicity-modulation, i.e. relative sensitivity or resistance.

### Requirement of autophagy to survive after MMS-induced damage

**Figure 3:**
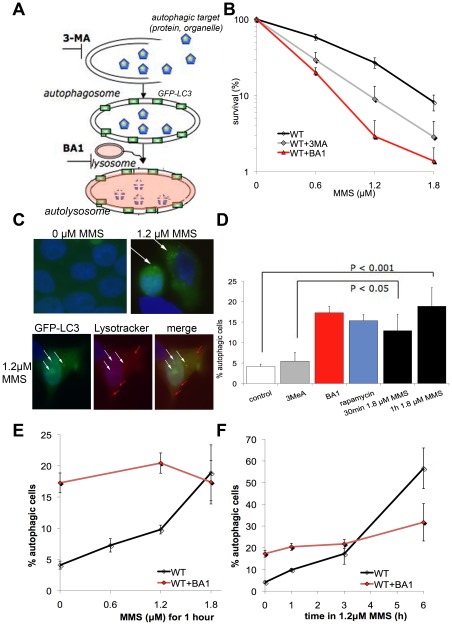
Response to MMS relies on autophagy. A) Model for induction and inhibition of autophagy. 3-methyladenine (3MeA) inhibits the formation of autophagosomes and bafiloycin A1 inhibits the acidification of the lysosomes leading to an accumulation of autophagosomes. LC3 is a marker of autophagosomes, here was tagged with GFP. B) Inhibition of autophagy decreases survival, both with 3MeA and BA1. C) Autophagy as seen by the formation of LC3-GFP-puncta showing autophagosomes in treated cells (top panel). A subset of the LC3-GFP-puncta co-stain (white arrows) with the acidic vesicles labeled by Lysotracker Red (white and red arrows) (bottom panel). D) Significant induction of autophagy after MMS treatment (1.2 µM). E–F) MMS induces autophagy in a dose and time dependent manner, whereas accumulation of autophagosomes by BA1 is not affected by MMS.

The toxicity-modulation results (Figure 2B) revealed that cells lacking the vesicle proteins ZFYVE20, ATP6V1D and VPS16 became sensitive to MMS, suggesting an involvement of early and late endosomal pathways in damage recovery. The late endosomal vesicle transport intersects with the autophagic pathway, and we set out to characterize the role of autophagy after damage by DNA damaging agents. During the autophagic process, cellular components are engulfed in autophagosomes with LC3-molecules on the surface. These autophagosomes are fused with acidic lysosomes to form autolysosomes where the engulfed components are broken down and possibly recycled (Figure 3A). To determine the significance of autophagy after DNA damage, we studied the effect of inhibiting autophagy in wild-type 293T cells. A chemical inhibitor of the early steps of autophagy (3-methyladenine, 3MeA) and an inhibitor of the late steps (Bafilomycin A1, BA1) were used (Figure 3A). BA1 inhibits autophagic completion and leads to accumulation of late autophagic vesicles. Wild-type cells were incubated in the presence of an autophagy inhibitor two hours prior to the one hour treatment with the damaging agent. The survival after MMS was severely diminished by the reduced autophagy mediated by both inhibitors (Figure 3B), indicating that autophagy is needed for the cells to recover after MMS exposure. Significant sensitization of the cells were observed after treatment with 4-NQO and t-BuOOH, although less pronounced compared to MMS (Figure S2). This finding indicate a general requirement for autophagy after cellular treatment with DNA damaging agents to rescue the exposed cells.

In an attempt to study the dynamics of autophagy induction, cells were transfected with GFP-tagged LC3 and followed during 6 hours. LC3 accumulates in the autophagosomes [32] and these GFP-labelled autophagosomes can be visualized as puncta using a fluorescent microscope. To further study the autophagic flux, we also followed the progression of autophagy by incubation of the cells with Lysotracker, which will stain acidic compartments such as lysosomes and autolysosomes. Cells that contained >5 visible puncta were scored as autophagic cells (Figure 3C). A subset of the LC3-positive autophagosomes (stained green) fuse with lysosomes (red) to make the autolysosomes (yellow), in both untreated and treated cells. This observation is consistent with previous studies of 293 cells [33], [34]. Low frequencies of autophagic cells were found in the control cultures with and without 3MeA. Cells were treated with BA1 to inhibit late steps of autophagy and thus trap the cells with induced but not completed autophagy, or with the known autophagy-inducer rapamycin. Both treatments induced 3–4-fold higher levels of autophagic cells. MMS treatment induced autophagy to the same extent (Figure 3D). Further analysis of the dose-dependencies of autophagy induction revealed a robust dose and time response for MMS. After pretreatment of the cells with inhibitor BA1, the percentage of cells with induced autophagy did not further increase upon subsequent MMS exposure (Figure 3E, F).

**Figure 4:**
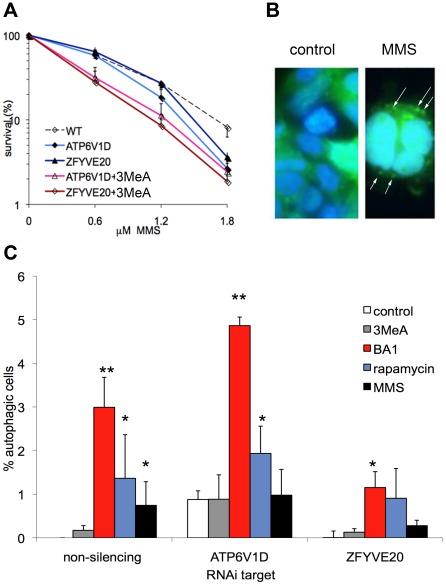
Autophagic response to MMS is modulated by ATP6V1D and ZFYVE20. A) Inhibition of autophagy further sensitizes the cells that have been depleted of ZFYVE20 and APT6V1D to MMS. B) The formation of autophagosomes after MMS treatment is visible in a background of cytoplasmic GFP. C) MMS induces autophagy in cells that is dependent on ZFYVE20. The statistical significance of the difference between each condition and its untreated control is indicated by asterisks (* p<0.05, ** p<0.01).

We then sought to elucidate the autophagy-related role of the proteins ATP6V1D and ZFYVE20. Cells depleted for ATP6V1D and ZFYVE20 were sensitive to MMS. Autophagy inhibitor 3MeA further sensitized both depleted cell lines at low loses of MMS (Figure 4A). The study of LC3 puncta was disadvantaged by the fact that the cells already expressed GFP to some level and therefore had a uniform cytoplasmic background of GFP. However, after transfection with the LC3-GFP construct, cells with clearly defined puncta within the GFP background could be scored (Figure 4B). As a consequence of the background GFP-levels, the percentages of identifiable autophagy-positive cells were lower in these cells (Figure 4C). The cell lines depleted of ATP6V1D and ZFYVE20 were both sensitive to MMS, and neither cell line was able to significantly induce autophagy over background levels following MMS treatment. Reduced levels of ATP6V1D lead to an accumulation of autophagosomes, suggesting that ATP6V1D is involved in the late steps of MMS-induced autophagy, in the clearance of the autophagosomes. However, reduced levels of ZFYVE20 resulted in a lower percentage of autophagic cells in the treated cultures, suggesting that ZFYVE20 is involved in the early steps of MMS-induced autophagy, such as the formation of the autophagosomes.

### Damage sensitivity and chromatin remodeling

Many targets in our screen affect the structural status of chromatin. These include histone modifiers such as a histone ubiquitin ligase (RNF20) and components of complexes changing the acetylation status of histones (TADA2A, SIN3A and HDAC6). TOP3A creates transient single stranded DNA breaks that will alter the topology of chromatin, and POLR2D is a subunit of RNA polymerase II. Reduced cellular levels of these proteins lead to increased (SIN3A, RNF20, TOP3A, POLR2D) or decreased (TADA2A, HDAC6) survival after 4-NQO exposure compared to survival of control cells. The bulky lesions induced by 4-NQO [7], [8] presumably cause conformational changes in chromatin, possibly explaining the requirement of chromatin modifiers for survival.

### Other proteins and functions

The telomere specific proteins tested in this study (TERF1, TERF2, ACD and POT1) all resulted in some cellular sensitivity to MMS when depleted, arguing that the enrichment for the ‘telomere maintenance’ term in the yeast screen is caused by the need to maintain intact telomeres for survival, in addition to the fact that the yeast telomere maintenance proteins also have an active role in DNA repair. RNA degradation was represented in this study by LSM1, EXOSC10, CDC40 and NCBP2. Depletion of these proteins led to 4NQO resistance (CDC40 and LSM1) or no visible phenotype (EXOSC10 and NCBP2). The rest of the targeted proteins form a mosaic of different known or unknown functions. Interestingly, the signaling protein CTDNEP1 (the homolog of yeast Nem1, YHR004C) [35] is one of the few proteins that lead to cellular sensitivity to t-BuOOH when depleted, relative to WT. XPA is the only tested protein with a clearly defined role in DNA repair. It was selected as a positive control as it is known that cells lacking the nucleotide excision repair component XPA are sensitive to 4-NQO [36]. We confirmed this finding, as the cell line deficit in XPA is one of the most 4-NQO sensitive in this screen.

## Discussion

‘DNA damaging agents’ cause damage to numerous cellular molecules and do not only damage DNA. In response to treatment with these agents, cells modulate the expression levels of genes in several different pathways. The results of this study show that many proteins and pathways are needed for recovery from specific types of damage. Previously, results from genome-wide RNAi screens have suggested the involvement of a vast repertoire of DNA damage recovery proteins. Among these are proteins involved in mRNA processing, chromatin binding and Charcot Marie Tooth-disease [19], [22], [23]. Here, as an alternative to genome-wide RNAi screens, we have implemented a focused approach where we take advantage of previous results from model organisms, such as *S. cerevisiae*, and extensive knowledge of protein-protein interactions and interactomes.

Among the human homologs of toxicity-modulating yeast proteins, 25 of the 34 proteins were found to be toxicity-modulating in human cells. The three DNA damaging agents, MMS, 4-NQO and t-BuOOH, revealed distinct toxicity profiles with proteins specifically conferring resistance or sensitivity to at least one of the three agents. Deficiency for only one of the proteins, the largely uncharacterized suppressor of actin mutations 1-like SACM1L [37], modulated toxicity for all three damaging agents. SACM1L is a phosphatase, regulating Golgi morphology [38], [39]; interestingly, deletion of the yeast homolog of this protein caused cellular sensitivity to all four damaging agents tested in the yeast screen [13].

One striking result of our study is that we have identified many proteins that, when their mRNA levels are reduced, cause relative resistance to the DNA damaging agents, even though the proteins were selected based on the corresponding yeast deletion strains being sensitive to the agents. This observation was particularly noteworthy after exposure to 4-NQO for the members of histone modifier complexes, RNF20, SIN3A, CDC40, topoisomerase TOP3A and RNA polymerase subunit POLR2D. Possibly, this is a reflection of differential maintenance of chromatin structure in mammals versus *S. cerevisiae*. While the discrepancy is puzzling, it has been shown previously that even within the same organism, cells of different origin can display distinctive, even opposite, phenotypes after being exposed to damaging agents [40], [41], [42], [43]. One dramatic example is that while mouse ES cells deficient in the Aag glycosylase are MMS sensitive, relative to WT, myeloid bone marrow cells and retinal rods and cones deficient in the same enzyme are extremely MMS resistant [43], [44].

Vesicle transporters were among the unexpected toxicity-modulators in the yeast gene deletion screen. The endosomal vesicle transport, especially the late endosomal/lysosomal transport, is used for degradation of biomaterial, a process that intersects with the pathway of autophagy. This group of proteins is highly conserved between yeast and humans. Interestingly, the classical autophagy proteins (ATG1-ATG31) were not overrepresented among the sensitive yeast deletion strains, suggesting an alternative autophagy-like path taken after treatment with DNA damaging agents. Processing by autophagosomes/lysosomes and proteasome are two ways to clear the cell of proteins and other biomaterial. The autophagosomes can be generated from the cytoplasm but can also be derived from the trans-Golgi, when cells are exposed to the topoisomerase II inhibitor etoposide [45]. The previously described role of autophagy in DNA damage response has usually been linked to the cellular death program, as several genotoxic agents have been shown to induce autophagic cell death [46], [47], [48], [49]. In contrast, this study suggests that cells escape cell death by induction of autophagy, because when autophagy is reduced, cells are more sensitive to MMS. This is supported by other recent studies that show that damage can also lead to non-lethal autophagy [45], [50]. The study of autophagy is also important from a clinical viewpoint because autophagy has been shown to suppress tumorigenesis [51], as well as clearing cells that contain protein aggregates such as those formed in Huntinton's disease [52].

Here, we have confirmed that telomere-specific proteins are needed for cells to recover after treatment DNA damaging agents. Telomere proteins in yeast are also involved in DNA damage repair, but here we have shown that specific loss of telomere maintenance, by reducing the protein levels of members of the shelterin complex, results in sensitivity to alkylating damage. We have also identified new toxicity-modulating proteins involved in chromatin modification. Previously, it was known that another Ada2-homolog, TADA2B a mammalian paralog of TADA2A, is needed in the cellular response to UV irradiation. This adaptor protein is part of the STAGA (homologous to SAGA in yeast) histone acetylation complex and is required for transcription of p53 responsive elements after UV [53], [54]. TADA2A on the other hand is a component of the similar histone acetylation complex PCAF, whose activity was recently implicated in the p53 pathway [55]. The Ada2 homologs have also been found in H2B deubiquitination complexes. Ubiquitination of H2B is performed by the ubiquitin ligase RNF20, also identified as a toxicity-modulator in this study. Interestingly, a component involved in the deubiquitination of H2B (TADAD2A) has the reverse toxicity-modulation compared to an H2B ubiquitin ligase (RNF20). Other studies have shown that depletion of RNF20 inhibits both G_1_ arrest and apoptosis, but stimulates tumor advancement; its promotor is often hypermethylated in tumors [56], [57]. Further, RNF20 ser-522 has been identified as an ATM/ATR phosphorylation substrate after exposure to ionizing radiation [57]. Depletion of the human RNF20 paralog RNF40 was recently shown to stimulate cell growth and cell migration [58]. RNF20/RNF40-mediated ubiquitination of H2B is a prerequisite for RNA PolII transcription, possibly explaining the observed similarity in toxicity-modulation between RNF20 and RNA PolII subunit D (POLR2D) (Figure 2).

### Conclusions

Based on yeast orthology and conserved network structures, we have identified several human proteins necessary for recovery after cellular damage, among them components of autophagy and chromatin modifiers. Clearly, the functional relationships between yeast and human homologs are complex as the lack of some proteins conferred sensitivity in yeast cells but in human cells resulted in resistance as compared to their WT counterparts. Nevertheless, by studying the machinery that surrounds the core DNA repair proteins, we obtain a better understanding of the way cells respond to genotoxic insults. Most of the identified toxicity-modulating proteins have not been linked to DNA repair, cell cycle arrest or cell death and highlight the vast array of proteins that are involved in damage recovery after exposure to DNA damaging agents.

## Materials and Methods

### Bioinformatic analysis

Genome-wide yeast sensitivity data [13] was downloaded from http://genomicphenotyping.mit.edu/source2.html. Human orthologs to the yeast proteins were identified through Ensembl and Inparanoid. Orthologues of *S. cerevisiae* genes of interest in human, mouse, and yeast, were obtained from Ensembl49 (http://ensembl.org/) [59]. A merged human interactome by A. Garrow, Y. Adeleye and G. Warner [28] combines human interactions reported in IntAct, DIP, BIND and HPRD, in addition to papers by [60], [61]. The interactome was queried using Cytoscape 2.6 (http://cytoscape.org).

Human expression data was used from [29]. Genes with microarray expression values >100 were considered expressed.

### Cell culture

293T cells (ICLC catalog code: HTL04001, [62]) and their derivatives were cultured in Dulbecco's minimal essential media (Invitrogen) complemented with 10% fetal bovine serum, L-glutamine, 1% penicillin, and streptomycin. shRNAs expressed in a lentiviral plasmid (pGIPZ) were purchased from Open Biosystems. Three to nine clones were analyzed for mRNA levels, and the clone with the lowest residual mRNA concentration was subsequently used. Identity of shRNAs and sequences of qRT-PCR primers (Eurofin) are found in the Table S3. Knockdown cells were compared with 293T cells expressing a non-targeting shRNA (#RHS4346). Virus was generated in 293T cells using packaging plasmids psPAX2, pMD2.G (Addgene plasmid 12260 and 12259). Parental cell lines were infected with virus and stable clones selected using Puromycin (Invivogen). A few proteins were targeted by multiple shRNA constructs. After shRNA infection, most genes had residual levels below 30%, a few had 30–60% residual levels. For some targets, no reduction in mRNA levels could be detected even though all target plasmids were incorporated into the parental cells, as determined by the co-expression of GFP. Catalog numbers and primer sequences are available in Table S3.

### Colony forming assay

50–5,000 cells were seeded in 6-well plates and 16 hours later cells were washed with PBS and exposed in duplicates to 4-NQO, tBuOH, MMS (Sigma) in serum-free media. After one hour, drug-containing media was replaced by complete media and incubated for 6 days. Colonies were washed with cold PBS, dried overnight, fixed and stained with 0.25% Methylene blue in ethanol and counted.

### Calculation of toxicity-modulation

For a protein to be called toxicity-modulating, the survival of its corresponding cell line had to be significantly different from the non-silencing control cells (p<0.05, t-test) and had to be at least 20% more sensitive or resistant (+/− 0.26 in log2-space) than any of the control cells in the ‘noise region’ (the region created by the boundaries of the cell lines without targeted knock-down) at at least one dose-point. For a protein to be confer ‘high sensitivity’/‘high resistance’, the cellular survival had exceed 25% (+/− 0.32 in log2-space) at – at least – one dose-point.

### Autophagy detection

The plasmid EGFP-LC3 was purchased from Addgene (plasmid 11546) [63], and transfected into 293T cells. Autophagy was inhibited by addition of 0.1 uM Bafilomycin A1 (B-1080 from LC Laboratories, Woburn, MA) or 10 mM 3-Methyladenine (from Sigma-Aldrich, Louisville) two hours prior to as well as during treatment with the DNA damaging agent. For LysoTracker Red staining, the cells were treated with 50 nM LysoTracker Red DND-99 (Invitrogen) at 37°C for 30 min. Cells were fixed in 3.7% formaldehyde, and nuclei were stained with Prolong Gold with DAPI (Invitrogen).

## Supporting Information

Figure S1 **Survival of cell lines without significant RNA reduction.** **A**) mRNA levels of target transcripts that were not significantly reduced (ns – non-silencing clone). **B–C**) Survival curves of the control cell line expressing non-silencing shRNA (black), four cell lines with non-significant reduction of levels of the targeted RNA (grey), and additional cell lines with reduced levels TMLHE (red), TADA2A (blue) and TERF2 (green) after treatment with **B**) 4-NQO or **C**) MMS.
(TIF)

Figure S2 **The survival of exposed WT cells is diminished after inhibition of autophagy.** The cells were exposed to A) 4-NQO, and B) tBuOOH.
(TIF)

Table S1 **GO terms enriched in networks of toxicity modulating proteins.** Enrichment in human cells (S1a) is contrasted with yeast cells (S1b).
(PDF)

Table S2 **Summary of the human potential toxicity-modulating proteins.** The summary includes the described function in the cell and the yeast homologs, together with a toxicity-modulation summary of both yeast and human cells.
(PDF)

Table S3 **shRNA constructs and qRT-PCR primers.**
(PDF)
