# Supplementary figures and images for: Identification of Novel Human Damage Response Proteins Targeted through Yeast Orthology
Source: PLoS One. 2012 May 16;7(5):e37368. doi: 10.1371/journal.pone.0037368 (PMC3353887; doi:10.1371/journal.pone.0037368)

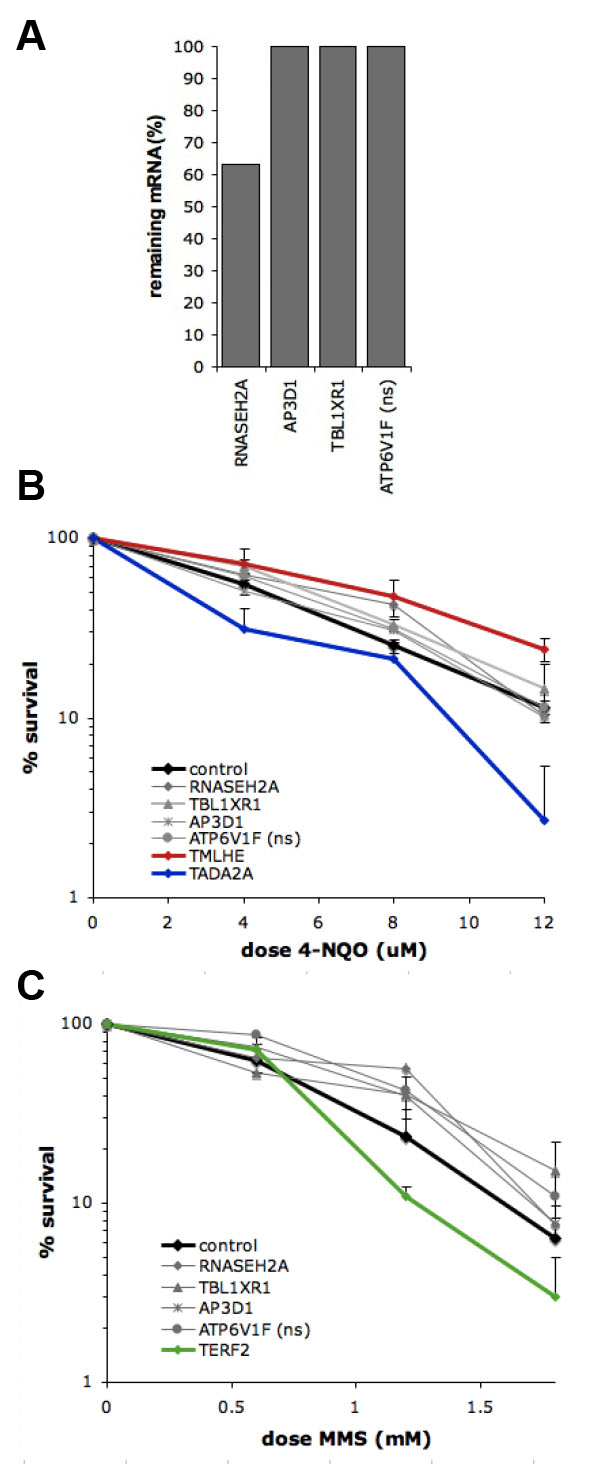

Supplement: Figure S1 — Survival of cell lines without significant RNA reduction. A) mRNA levels of target transcripts that were not significantly reduced (ns – non-silencing clone). B–C) Survival curves of the control cell line expressing non-silencing shRNA (black), four cell lines with non-significant reduction of levels of the targeted RNA (grey), and additional cell lines with reduced levels TMLHE (red), TADA2A (blue) and TERF2 (green) after treatment with B) 4-NQO or C) MMS. (TIF) [file pone.0037368.s001.tif]

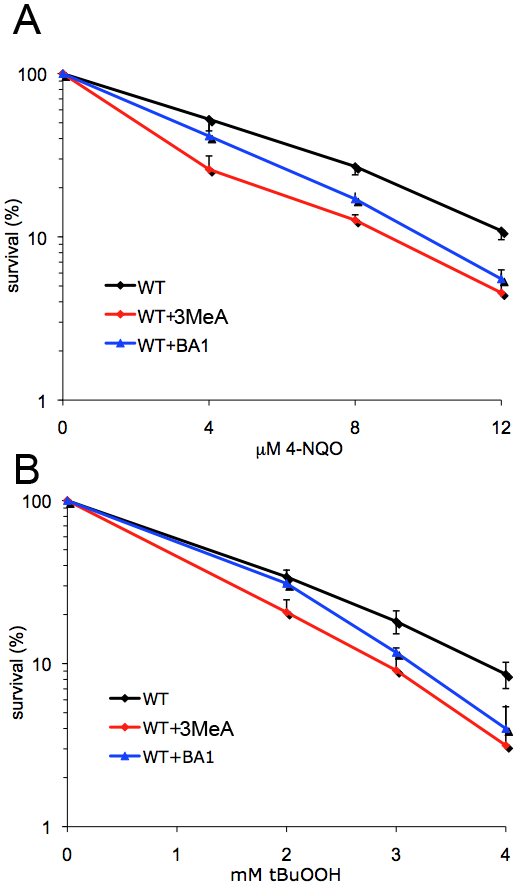

Supplement: Figure S2 — The survival of exposed WT cells is diminished after inhibition of autophagy. The cells were exposed to A) 4-NQO, and B) tBuOOH. (TIF) [file pone.0037368.s002.tif]
