# Supplementary material for: Identification of Novel Human Damage Response Proteins Targeted through Yeast Orthology
Source: PLoS One. 2012 May 16;7(5):e37368. doi: 10.1371/journal.pone.0037368 (PMC3353887; doi:10.1371/journal.pone.0037368)
Supplement: Table S1 — GO terms enriched in networks of toxicity modulating proteins. Enrichment in human cells (S1a) is contrasted with yeast cells (S1b). (PDF) [file pone.0037368.s003.pdf]

Tab le S1a Human Network

GO ID	Description	p-value	adjusted p-value (FDR)	representation in cluster (protein in cluster/cluster size, percentage)	total representation (protein in total/total size, percentage)
15992	proton transport	1.39E-16	1.32E-13	18/272 6.6%	66/14529 0.4%
6818	hydrogen transport	2.50E-16	1.32E-13	18/272 6.6%	68/14529 0.4%
8152	metabolic process	5.07E-15	1.78E-12	196/272 72.0%	7117/14529 48.9%
44237	cellular metabolic process	1.16E-14	3.04E-12	184/272 67.6%	6495/14529 44.7%
44238	primary metabolic process	1.53E-14	3.22E-12	181/272 66.5%	6348/14529 43.6%
6281	DNA repair	8.93E-13	1.57E-10	27/272 9.9%	267/14529 1.8%
6974	response to DNA damage stimulus	1.52E-11	2.28E-09	28/272 10.2%	324/14529 2.2%
6455	translational elongation	6.58E-11	8.66E-09	16/272 5.8%	102/14529 0.7%
46034	ATP metabolic process	1.55E-10	1.82E-08	11/272 4.0%	41/14529 0.2%
9259	ribonucleotide metabolic process	2.33E-10	2.45E-08	14/272 5.1%	80/14529 0.5%
9150	purine ribonucleotide metabolic process	4.78E-10	4.57E-08	13/272 4.7%	70/14529 0.4%
6091	energy	1.06E-09	9.31E-08	22/272 8.0%	243/14529 1.6%
6259	DNA metabolic process	2.08E-09	1.67E-07	30/272 11.0%	455/14529 3.1%
18106	peptidyl-histidine phosphorylation	2.22E-09	1.67E-07	5/272 1.8%	5/14529 0.0%
6163	purine nucleotide metabolic process	2.69E-09	1.89E-07	13/272 4.7%	80/14529 0.5%
9205	purine ribonucleoside triphosphate metabolic process	3.07E-09	1.90E-07	11/272 4.0%	53/14529 0.3%
9144	purine nucleoside triphosphate metabolic process	3.07E-09	1.90E-07	11/272 4.0%	53/14529 0.3%
6754	ATP biosynthetic process	3.48E-09	2.04E-07	9/272 3.3%	31/14529 0.2%
9199	process	3.80E-09	2.10E-07	11/272 4.0%	54/14529 0.3%
9260	ribonucleotide biosynthetic process	4.97E-09	2.62E-07	12/272 4.4%	69/14529 0.4%
7001	chromosome organization and biogenesis	6.89E-09	3.45E-07	27/272 9.9%	396/14529 2.7%
6325	establishment and/or maintenance of chromatin architecture	8.94E-09	4.10E-07	24/272 8.8%	322/14529 2.2%
6310	DNA recombination	8.95E-09	4.10E-07	13/272 4.7%	88/14529 0.6%
9152	purine ribonucleotide biosynthetic process	1.48E-08	6.48E-07	11/272 4.0%	61/14529 0.4%
9141	nucleoside triphosphate metabolic process	1.77E-08	7.44E-07	11/272 4.0%	62/14529 0.4%
6164	purine nucleotide biosynthetic process	2.96E-08	1.20E-06	11/272 4.0%	65/14529 0.4%
7145	meiotic recombination	3.10E-08	1.21E-06	7/272 2.5%	19/14529 0.1%
18202	peptidyl-histidine modification	4.52E-08	1.68E-06	5/272 1.8%	7/14529 0.0%
15031	protein transport	4.62E-08	1.68E-06	35/272 12.8%	676/14529 4.6%
45184	establishment of protein localization	4.80E-08	1.68E-06	35/272 12.8%	677/14529 4.6%
43574	peroxisomal transport	6.26E-08	2.13E-06	6/272 2.2%	13/14529 0.0%
7031	peroxisome organization and biogenesis	6.93E-08	2.28E-06	7/272 2.5%	21/14529 0.1%
9206	purine ribonucleoside triphosphate biosynthetic process	8.00E-08	2.48E-06	9/272 3.3%	43/14529 0.2%
9145	purine nucleoside triphosphate biosynthetic process	8.00E-08	2.48E-06	9/272 3.3%	43/14529 0.2%
9201	ribonucleoside triphosphate biosynthetic process	9.89E-08	2.98E-06	9/272 3.3%	44/14529 0.3%
44249	cellular biosynthetic process	1.58E-07	4.62E-06	47/272 17.2%	1129/14529 7.7%
9142	process	1.81E-07	5.03E-06	9/272 3.3%	47/14529 0.3%
6333	chromatin assembly or disassembly	1.82E-07	5.03E-06	15/272 5.5%	153/14529 1.0%
8104	protein localization	1.86E-07	5.03E-06	36/272 13.2%	749/14529 5.1%
6260	DNA replication	1.91E-07	5.03E-06	17/272 6.2%	198/14529 1.3%
6996	organelle organization and biogenesis	3.07E-07	7.90E-06	47/272 17.2%	1155/14529 7.9%
6302	double-strand break repair	3.28E-07	8.23E-06	8/272 2.9%	37/14529 0.2%
44262	cellular carbohydrate metabolic process	3.82E-07	9.35E-06	20/272 7.3%	283/14529 1.9%
55086	nucleobase, nucleoside and nucleotide metabolic process	5.78E-07	1.38E-05	17/272 6.2%	214/14529 1.4%
33036	macromolecule localization	6.93E-07	1.62E-05	36/272 13.2%	791/14529 5.4%
7126	meiosis	7.57E-07	1.70E-05	10/272 3.6%	71/14529 0.4%
51327	M phase of meiotic cell cycle	7.57E-07	1.70E-05	10/272 3.6%	71/14529 0.4%
15980	energy derivation by oxidation of organic compounds	8.12E-07	1.78E-05	11/272 4.0%	89/14529 0.6%
51321	meiotic cell cycle	9.86E-07	2.06E-05	10/272 3.6%	73/14529 0.5%
9117	nucleotide metabolic process	9.96E-07	2.06E-05	16/272 5.8%	198/14529 1.3%
6753	nucleoside phosphate metabolic process	9.96E-07	2.06E-05	16/272 5.8%	198/14529 1.3%
7127	meiosis I	1.05E-06	2.13E-05	7/272 2.5%	30/14529 0.2%

19752	carboxylic acid metabolic process	1.17E-06	2.33E-05	26/272 9.5%	479/14529 3.2%
9058	biosynthetic process	1.27E-06	2.48E-05	89/272 32.7%	2977/14529 20.4%
16043	biogenesis	1.32E-06	2.48E-05	59/272 21.6%	1688/14529 11.6%
6082	organic acid metabolic process	1.32E-06	2.48E-05	26/272 9.5%	482/14529 3.3%
6625	protein targeting to peroxisome	1.58E-06	2.91E-05	5/272 1.8%	12/14529 0.0%
6416	translation	2.26E-06	4.10E-05	25/272 9.1%	465/14529 3.2%
6338	chromatin remodeling	3.16E-06	5.62E-05	8/272 2.9%	49/14529 0.3%
5977	glycogen metabolic process	3.20E-06	5.62E-05	7/272 2.5%	35/14529 0.2%
6006	glucose metabolic process	3.52E-06	6.07E-05	11/272 4.0%	103/14529 0.7%
5996	monosaccharide metabolic process	3.60E-06	6.12E-05	13/272 4.7%	146/14529 1.0%
6073	glucan metabolic process	3.91E-06	6.54E-05	7/272 2.5%	36/14529 0.2%
6139	nucleobase, nucleoside, nucleotide and nucleic acid metabolic process	4.07E-06	6.70E-05	83/272 30.5%	2786/14529 19.1%
9165	nucleotide biosynthetic process	4.26E-06	6.90E-05	12/272 4.4%	126/14529 0.8%
51234	establishment of localization	5.10E-06	8.14E-05	78/272 28.6%	2578/14529 17.7%
6810	transport	5.44E-06	8.55E-05	77/272 28.3%	2538/14529 17.4%
6263	DNA-dependent DNA replication	6.68E-06	1.03E-04	9/272 3.3%	71/14529 0.4%
43284	biopolymer biosynthetic process	9.20E-06	1.40E-04	62/272 22.7%	1921/14529 13.2%
6112	energy reserve metabolic process	9.73E-06	1.46E-04	7/272 2.5%	41/14529 0.2%
6476	protein amino acid deacetylation	1.14E-05	1.69E-04	5/272 1.8%	17/14529 0.1%
43170	macromolecule metabolic process	1.29E-05	1.88E-04	133/272 48.8%	5281/14529 36.3%
16558	protein import into peroxisome matrix	1.41E-05	2.03E-04	4/272 1.4%	9/14529 0.0%
16192	vesicle-mediated transport	1.58E-05	2.25E-04	23/272 8.4%	455/14529 3.1%
8151	cellular process	1.79E-05	2.51E-04	238/272 87.5%	11268/14529 77.5%
16568	chromatin modification	1.96E-05	2.72E-04	14/272 5.1%	196/14529 1.3%
16575	histone deacetylation	3.58E-05	4.89E-04	4/272 1.4%	11/14529 0.0%
15672	monovalent inorganic cation transport	3.72E-05	5.03E-04	18/272 6.6%	322/14529 2.2%
18193	peptidyl-amino acid modification	3.89E-05	5.18E-04	9/272 3.3%	88/14529 0.6%
9059	macromolecule biosynthetic process	4.39E-05	5.78E-04	72/272 26.4%	2462/14529 16.9%
51179	localization	5.07E-05	6.59E-04	81/272 29.7%	2882/14529 19.8%
15985	energy coupled proton transport, down electrochemical gradient	5.66E-05	7.18E-04	5/272 1.8%	23/14529 0.1%
15986	ATP synthesis coupled proton transport	5.66E-05	7.18E-04	5/272 1.8%	23/14529 0.1%
43101	purine salvage	6.31E-05	7.91E-04	3/272 1.1%	5/14529 0.0%
19318	hexose metabolic process	6.50E-05	8.05E-04	11/272 4.0%	140/14529 0.9%
34220	transmembrane ion transport	8.66E-05	1.06E-03	5/272 1.8%	25/14529 0.1%
5975	carbohydrate metabolic process	9.99E-05	1.21E-03	21/272 7.7%	445/14529 3.0%
44264	cellular polysaccharide metabolic process	1.11E-04	1.33E-03	7/272 2.5%	59/14529 0.4%
43283	biopolymer metabolic process	1.36E-04	1.61E-03	105/272 38.6%	4111/14529 28.2%
5976	polysaccharide metabolic process	1.37E-04	1.61E-03	7/272 2.5%	61/14529 0.4%
6298	mismatch repair	1.82E-04	2.08E-03	5/272 1.8%	29/14529 0.1%
45005	maintenance of fidelity during DNA-dependent DNA replication	1.82E-04	2.08E-03	5/272 1.8%	29/14529 0.1%
16197	endosome transport	2.04E-04	2.30E-03	6/272 2.2%	46/14529 0.3%
15988	energy coupled proton transport, against electrochemical gradient	2.15E-04	2.41E-03	3/272 1.1%	7/14529 0.0%
6631	fatty acid metabolic process	2.38E-04	2.64E-03	11/272 4.0%	162/14529 1.1%
31497	chromatin assembly	3.09E-04	3.38E-03	9/272 3.3%	115/14529 0.7%
6950	response to stress	3.25E-04	3.52E-03	41/272 15.0%	1258/14529 8.6%
32787	monocarboxylic acid metabolic process	3.27E-04	3.52E-03	13/272 4.7%	225/14529 1.5%
38	process	3.39E-04	3.53E-03	3/272 1.1%	8/14529 0.0%
45843	negative regulation of striated muscle development	3.39E-04	3.53E-03	3/272 1.1%	8/14529 0.0%
19321	pentose metabolic process	3.39E-04	3.53E-03	3/272 1.1%	8/14529 0.0%
6855	multidrug transport	3.49E-04	3.61E-03	2/272 0.7%	2/14529 0.0%
6007	glucose catabolic process	4.95E-04	4.98E-03	6/272 2.2%	54/14529 0.3%
6563	L-serine metabolic process	5.01E-04	4.98E-03	3/272 1.1%	9/14529 0.0%
43094	metabolic compound salvage	5.01E-04	4.98E-03	3/272 1.1%	9/14529 0.0%
48635	negative regulation of muscle development	5.01E-04	4.98E-03	3/272 1.1%	9/14529 0.0%
16570	histone modification	5.92E-04	5.82E-03	5/272 1.8%	37/14529 0.2%
5978	glycogen biosynthetic process	7.06E-04	6.64E-03	3/272 1.1%	10/14529 0.0%
46128	purine ribonucleoside metabolic process	7.06E-04	6.64E-03	3/272 1.1%	10/14529 0.0%
9250	glucan biosynthetic process	7.06E-04	6.64E-03	3/272 1.1%	10/14529 0.0%
42278	purine nucleoside metabolic process	7.06E-04	6.64E-03	3/272 1.1%	10/14529 0.0%
42147	retrograde transport, endosome to Golgi	7.06E-04	6.64E-03	3/272 1.1%	10/14529 0.0%
22403	cell cycle phase	7.15E-04	6.66E-03	15/272 5.5%	308/14529 2.1%
55085	transmembrane transport	7.58E-04	6.94E-03	5/272 1.8%	39/14529 0.2%

16569	covalent chromatin modification	7.58E-04	6.94E-03	5/272 1.8%	39/14529 0.2%
10324	membrane invagination	8.28E-04	7.45E-03	10/272 3.6%	159/14529 1.0%
16193	endocytosis	8.28E-04	7.45E-03	10/272 3.6%	159/14529 1.0%
6732	coenzyme metabolic process	9.41E-04	8.40E-03	9/272 3.3%	134/14529 0.9%
7588	excretion	9.57E-04	8.47E-03	5/272 1.8%	41/14529 0.2%
19	regulation of mitotic recombination	1.03E-03	9.00E-03	2/272 0.7%	3/14529 0.0%
6654	phosphatidic acid biosynthetic process	1.03E-03	9.00E-03	2/272 0.7%	3/14529 0.0%
44275	cellular carbohydrate catabolic process	1.06E-03	9.16E-03	7/272 2.5%	85/14529 0.5%
19320	hexose catabolic process	1.13E-03	9.69E-03	6/272 2.2%	63/14529 0.4%
46365	monosaccharide catabolic process	1.23E-03	1.04E-02	6/272 2.2%	64/14529 0.4%
9119	ribonucleoside metabolic process	1.26E-03	1.06E-02	3/272 1.1%	12/14529 0.0%
6066	alcohol metabolic process	1.37E-03	1.14E-02	14/272 5.1%	295/14529 2.0%
6323	DNA packaging	1.42E-03	1.17E-02	9/272 3.3%	142/14529 0.9%
16044	membrane organization and biogenesis	1.60E-03	1.30E-02	13/272 4.7%	267/14529 1.8%
6268	DNA unwinding during replication	1.61E-03	1.30E-02	3/272 1.1%	13/14529 0.0%
724	double-strand break repair via homologous recombination	1.61E-03	1.30E-02	3/272 1.1%	13/14529 0.0%
725	recombinational repair	1.61E-03	1.30E-02	3/272 1.1%	13/14529 0.0%
44260	cellular macromolecule metabolic process	1.62E-03	1.30E-02	73/272 26.8%	2820/14529 19.4%
16052	carbohydrate catabolic process	1.69E-03	1.33E-02	7/272 2.5%	92/14529 0.6%
46164	alcohol catabolic process	1.69E-03	1.33E-02	6/272 2.2%	68/14529 0.4%
6166	purine ribonucleoside salvage	2.04E-03	1.57E-02	2/272 0.7%	4/14529 0.0%
43174	nucleoside salvage	2.04E-03	1.57E-02	2/272 0.7%	4/14529 0.0%
46473	phosphatidic acid metabolic process	2.04E-03	1.57E-02	2/272 0.7%	4/14529 0.0%
32392	DNA geometric change	2.50E-03	1.88E-02	3/272 1.1%	15/14529 0.1%
32508	DNA duplex unwinding	2.50E-03	1.88E-02	3/272 1.1%	15/14529 0.1%
16202	regulation of striated muscle development	2.50E-03	1.88E-02	3/272 1.1%	15/14529 0.1%
46907	intracellular transport	2.56E-03	1.91E-02	23/272 8.4%	649/14529 4.4%
279	M phase	2.97E-03	2.20E-02	12/272 4.4%	253/14529 1.7%
48634	regulation of muscle development	3.03E-03	2.23E-02	3/272 1.1%	16/14529 0.1%
6812	cation transport	3.13E-03	2.29E-02	19/272 6.9%	505/14529 3.4%
6119	oxidative phosphorylation	3.18E-03	2.31E-02	6/272 2.2%	77/14529 0.5%
45744	negative regulation of G-protein coupled receptor protein signaling pathway	3.36E-03	2.33E-02	2/272 0.7%	5/14529 0.0%
6312	mitotic recombination	3.36E-03	2.33E-02	2/272 0.7%	5/14529 0.0%
9203	process	3.36E-03	2.33E-02	2/272 0.7%	5/14529 0.0%
6200	ATP catabolic process	3.36E-03	2.33E-02	2/272 0.7%	5/14529 0.0%
9207	purine ribonucleoside triphosphate catabolic process	3.36E-03	2.33E-02	2/272 0.7%	5/14529 0.0%
9146	purine nucleoside triphosphate catabolic process	3.36E-03	2.33E-02	2/272 0.7%	5/14529 0.0%
7025	beta-tubulin folding	3.36E-03	2.33E-02	2/272 0.7%	5/14529 0.0%
6334	nucleosome assembly	3.39E-03	2.33E-02	7/272 2.5%	104/14529 0.7%
51186	cofactor metabolic process	3.62E-03	2.46E-02	9/272 3.3%	163/14529 1.1%
22607	cellular component assembly	3.62E-03	2.46E-02	9/272 3.3%	163/14529 1.1%
9154	purine ribonucleotide catabolic process	4.98E-03	3.36E-02	2/272 0.7%	6/14529 0.0%
9411	response to UV	5.38E-03	3.61E-02	4/272 1.4%	38/14529 0.2%
6301	postreplication repair	6.89E-03	4.54E-02	2/272 0.7%	7/14529 0.0%
60070	Wnt receptor signaling pathway through beta-catenin	6.89E-03	4.54E-02	2/272 0.7%	7/14529 0.0%
9650	UV protection	6.89E-03	4.54E-02	2/272 0.7%	7/14529 0.0%
30097	hemopoiesis	7.06E-03	4.61E-02	7/272 2.5%	119/14529 0.8%

Table S1b Yeast Network

GO ID	Description	p-value	adjusted p-value (FDR)	representation in cluster (protein in cluster/cluster size, percentage)	representation (protein in total/total size, percentage)
7001	chromosome organization and biogenesis	1.21E-27	2.18E-24	172/1172 14.6%	398/5819 6.8%
6974	response to DNA damage stimulus	1.62E-26	1.47E-23	133/1172 11.3%	278/5819 4.7%
65007	biological regulation	1.07E-24	6.46E-22	493/1172 42.0%	1722/5819 29.5%
6950	response to stress	9.90E-24	4.48E-21	229/1172 19.5%	632/5819 10.8%
51869	response to stimulus	5.17E-23	1.87E-20	312/1172 26.6%	966/5819 16.6%
50791	regulation of biological process	9.78E-21	2.95E-18	430/1172 36.6%	1500/5819 25.7%
45934	nucleoside, nucleotide and nucleic acid metabolic process	3.67E-19	9.49E-17	101/1172 8.6%	217/5819 3.7%
6325	establishment and/or maintenance of chromatin architecture	5.60E-19	1.27E-16	114/1172 9.7%	260/5819 4.4%
6281	DNA repair	6.65E-19	1.34E-16	104/1172 8.8%	228/5819 3.9%
51244	regulation of cellular process	1.13E-18	1.96E-16	413/1172 35.2%	1457/5819 25.0%
19219	nucleotide and nucleic acid metabolic process	1.19E-18	1.96E-16	234/1172 19.9%	703/5819 12.0%
9892	negative regulation of metabolic process	2.57E-18	3.88E-16	110/1172 9.3%	251/5819 4.3%
31324	process	5.92E-18	8.25E-16	109/1172 9.3%	250/5819 4.2%
48519	negative regulation of biological process	6.81E-18	8.81E-16	122/1172 10.4%	294/5819 5.0%
16568	chromatin modification	7.48E-18	9.03E-16	103/1172 8.7%	231/5819 3.9%
48523	negative regulation of cellular process	5.13E-17	5.81E-15	119/1172 10.1%	290/5819 4.9%
8151	cellular process	6.28E-16	6.69E-14	1047/1172 89.3%	4748/5819 81.5%
19222	regulation of metabolic process	1.01E-15	1.02E-13	333/1172 28.4%	1154/5819 19.8%
45910	negative regulation of DNA recombination	5.61E-15	5.34E-13	33/1172 2.8%	44/5819 0.7%
51053	process	7.29E-15	6.48E-13	39/1172 3.3%	58/5819 0.9%
10526	negative regulation of transposition, RNA-mediated	7.87E-15	6.48E-13	31/1172 2.6%	40/5819 0.6%
10529	negative regulation of transposition	7.87E-15	6.48E-13	31/1172 2.6%	40/5819 0.6%
51252	regulation of RNA metabolic process	9.48E-15	7.47E-13	200/1172 17.0%	614/5819 10.5%
31323	regulation of cellular metabolic process	1.86E-14	1.40E-12	316/1172 26.9%	1101/5819 18.9%
6355	regulation of transcription, DNA-dependent	5.02E-14	3.63E-12	195/1172 16.6%	603/5819 10.3%
18	regulation of DNA recombination	7.03E-14	4.90E-12	35/1172 2.9%	51/5819 0.8%
10525	regulation of transposition, RNA-mediated	8.07E-14	5.22E-12	31/1172 2.6%	42/5819 0.7%
10528	regulation of transposition	8.07E-14	5.22E-12	31/1172 2.6%	42/5819 0.7%
45449	regulation of transcription	1.04E-13	6.46E-12	201/1172 17.1%	631/5819 10.8%
51052	regulation of DNA metabolic process	4.76E-13	2.87E-11	50/1172 4.2%	93/5819 1.5%
6302	double-strand break repair	9.49E-13	5.55E-11	37/1172 3.1%	59/5819 1.0%
43687	post-translational protein modification	1.10E-12	6.22E-11	153/1172 13.0%	454/5819 7.8%
10605	negative regulation of macromolecule metabolic process	3.28E-12	1.80E-10	88/1172 7.5%	219/5819 3.7%
51321	meiotic cell cycle	4.66E-12	2.35E-10	75/1172 6.3%	176/5819 3.0%
51327	M phase of meiotic cell cycle	4.66E-12	2.35E-10	75/1172 6.3%	176/5819 3.0%
7126	meiosis	4.66E-12	2.35E-10	75/1172 6.3%	176/5819 3.0%
6259	DNA metabolic process	1.50E-11	7.34E-10	146/1172 12.4%	440/5819 7.5%
60255	process	1.87E-11	8.93E-10	297/1172 25.3%	1071/5819 18.4%
10468	regulation of gene expression	3.69E-11	1.71E-09	282/1172 24.0%	1011/5819 17.3%
16481	negative regulation of transcription	4.44E-11	1.96E-09	70/1172 5.9%	166/5819 2.8%
10629	negative regulation of gene expression	4.44E-11	1.96E-09	70/1172 5.9%	166/5819 2.8%
22403	cell cycle phase	4.71E-11	2.03E-09	136/1172 11.6%	407/5819 6.9%
9890	negative regulation of biosynthetic process	4.86E-11	2.05E-09	77/1172 6.5%	190/5819 3.2%
7034	vacuolar transport	8.01E-11	3.30E-09	56/1172 4.7%	122/5819 2.0%
9889	regulation of biosynthetic process	9.12E-11	3.67E-09	281/1172 23.9%	1014/5819 17.4%
726	non-recombinational repair	9.51E-11	3.74E-09	24/1172 2.0%	33/5819 0.5%
10558	negative regulation of macromolecule biosynthetic process	1.11E-10	4.29E-09	73/1172 6.2%	179/5819 3.0%
45892	negative regulation of transcription, DNA-dependent	1.37E-10	5.16E-09	66/1172 5.6%	156/5819 2.6%
10556	regulation of macromolecule biosynthetic process	1.77E-10	6.53E-09	277/1172 23.6%	1002/5819 17.2%
51253	process	1.91E-10	6.89E-09	66/1172 5.6%	157/5819 2.6%

279	M phase	1.94E-10	6.89E-09	108/1172 9.2%	307/5819 5.2%
6996	organelle organization and biogenesis	2.12E-10	7.39E-09	405/1172 34.5%	1579/5819 27.1%
6338	chromatin remodeling	5.24E-10	1.79E-08	63/1172 5.3%	150/5819 2.5%
16570	histone modification	1.78E-09	5.86E-08	44/1172 3.7%	92/5819 1.5%
16569	covalent chromatin modification	1.78E-09	5.86E-08	44/1172 3.7%	92/5819 1.5%
16043	biogenesis	4.63E-09	1.50E-07	487/1172 41.5%	1997/5819 34.3%
16458	gene silencing	5.12E-09	1.61E-07	47/1172 4.0%	104/5819 1.7%
22402	cell cycle process	5.15E-09	1.61E-07	142/1172 12.1%	457/5819 7.8%
6623	protein targeting to vacuole	6.63E-09	2.04E-07	33/1172 2.8%	62/5819 1.0%
65008	regulation of biological quality	9.90E-09	2.99E-07	121/1172 10.3%	377/5819 6.4%
6476	protein amino acid deacetylation	1.61E-08	4.79E-07	20/1172 1.7%	29/5819 0.4%
17035	response to drug	2.09E-08	6.10E-07	51/1172 4.3%	121/5819 2.0%
6350	transcription	3.51E-08	1.01E-06	173/1172 14.7%	598/5819 10.2%
44237	cellular metabolic process	4.07E-08	1.15E-06	829/1172 70.7%	3727/5819 64.0%
7049	cell cycle	4.33E-08	1.21E-06	165/1172 14.0%	566/5819 9.7%
6333	chromatin assembly or disassembly	5.51E-08	1.51E-06	51/1172 4.3%	124/5819 2.1%
8152	metabolic process	5.78E-08	1.56E-06	857/1172 73.1%	3878/5819 66.6%
31497	chromatin assembly	7.88E-08	2.10E-06	48/1172 4.0%	115/5819 1.9%
6464	protein modification process	1.38E-07	3.63E-06	199/1172 16.9%	720/5819 12.3%
6914	autophagy	1.41E-07	3.64E-06	81/1172 6.9%	236/5819 4.0%
6323	DNA packaging	1.62E-07	4.14E-06	52/1172 4.4%	131/5819 2.2%
725	recombinational repair	1.94E-07	4.89E-06	20/1172 1.7%	32/5819 0.5%
48869	cellular developmental process	6.27E-07	1.55E-05	105/1172 8.9%	338/5819 5.8%
724	double-strand break repair via homologous recombination	6.48E-07	1.57E-05	17/1172 1.4%	26/5819 0.4%
51651	maintenance of location in cell	6.48E-07	1.57E-05	17/1172 1.4%	26/5819 0.4%
45814	negative regulation of gene expression, epigenetic	8.15E-07	1.89E-05	39/1172 3.3%	92/5819 1.5%
6342	chromatin silencing	8.15E-07	1.89E-05	39/1172 3.3%	92/5819 1.5%
31507	heterochromatin formation	8.15E-07	1.89E-05	39/1172 3.3%	92/5819 1.5%
16575	histone deacetylation	9.48E-07	2.17E-05	16/1172 1.3%	24/5819 0.4%
42592	homeostatic process	1.03E-06	2.32E-05	75/1172 6.3%	223/5819 3.8%
7127	meiosis I	1.34E-06	3.00E-05	34/1172 2.9%	77/5819 1.3%
44238	primary metabolic process	1.79E-06	3.94E-05	773/1172 65.9%	3493/5819 60.0%
51235	maintenance of location	1.91E-06	4.16E-05	18/1172 1.5%	30/5819 0.5%
7154	cell communication	1.94E-06	4.17E-05	98/1172 8.3%	317/5819 5.4%
723	telomere maintenance	2.01E-06	4.18E-05	28/1172 2.3%	59/5819 1.0%
60249	anatomical structure homeostasis	2.01E-06	4.18E-05	28/1172 2.3%	59/5819 1.0%
32200	telomere organization and biogenesis	2.01E-06	4.18E-05	28/1172 2.3%	59/5819 1.0%
6303	double-strand break repair via nonhomologous end joining	2.14E-06	4.41E-05	16/1172 1.3%	25/5819 0.4%
40029	regulation of gene expression, epigenetic	2.28E-06	4.63E-05	41/1172 3.4%	102/5819 1.7%
16197	endosome transport	3.05E-06	6.14E-05	28/1172 2.3%	60/5819 1.0%
45324	late endosome to vacuole transport	4.33E-06	8.59E-05	19/1172 1.6%	34/5819 0.5%
45002	double-strand break repair via single-strand annealing	4.36E-06	8.59E-05	9/1172 0.7%	10/5819 0.1%
51179	localization	5.56E-06	1.08E-04	303/1172 25.8%	1225/5819 21.0%
74	regulation of cell cycle	6.37E-06	1.22E-04	56/1172 4.7%	160/5819 2.7%
32502	developmental process	6.41E-06	1.22E-04	131/1172 11.1%	462/5819 7.9%
32507	maintenance of protein location in cell	6.77E-06	1.28E-04	13/1172 1.1%	19/5819 0.3%
6468	protein amino acid phosphorylation	7.73E-06	1.44E-04	49/1172 4.1%	135/5819 2.3%
43284	biopolymer biosynthetic process	1.05E-05	1.94E-04	241/1172 20.5%	950/5819 16.3%
16310	phosphorylation	1.09E-05	1.99E-04	64/1172 5.4%	193/5819 3.3%
7145	meiotic recombination	1.15E-05	2.09E-04	26/1172 2.2%	57/5819 0.9%
6793	phosphorus metabolic process	1.53E-05	2.74E-04	85/1172 7.2%	278/5819 4.7%
30154	cell differentiation	1.54E-05	2.74E-04	63/1172 5.3%	191/5819 3.2%
43486	histone exchange	1.97E-05	3.43E-04	8/1172 0.6%	9/5819 0.1%
45053	protein retention in Golgi apparatus	1.97E-05	3.43E-04	9/1172 0.7%	11/5819 0.1%
43162	process via the multivesicular body pathway	2.34E-05	4.03E-04	12/1172 1.0%	18/5819 0.3%
6796	phosphate metabolic process	2.49E-05	4.25E-04	79/1172 6.7%	257/5819 4.4%
819	sister chromatid segregation	2.55E-05	4.31E-04	29/1172 2.4%	69/5819 1.1%
45185	maintenance of protein location	2.72E-05	4.56E-04	15/1172 1.2%	26/5819 0.4%
6605	protein targeting	3.42E-05	5.68E-04	70/1172 5.9%	223/5819 3.8%
42221	response to chemical stimulus	4.03E-05	6.63E-04	124/1172 10.5%	448/5819 7.6%
51641	cellular localization	4.78E-05	7.79E-04	175/1172 14.9%	672/5819 11.5%
7242	intracellular signaling cascade	6.15E-05	9.94E-04	60/1172 5.1%	187/5819 3.2%

33365	protein localization in organelle	6.46E-05	1.03E-03	9/1172 0.7%	12/5819 0.2%
34067	protein localization in Golgi apparatus	6.46E-05	1.03E-03	9/1172 0.7%	12/5819 0.2%
7021	tubulin complex assembly	6.61E-05	1.04E-03	6/1172 0.5%	6/5819 0.1%
51234	establishment of localization	7.23E-05	1.13E-03	285/1172 24.3%	1177/5819 20.2%
45003	double-strand break repair via synthesis-dependent strand annealing	7.87E-05	1.22E-03	11/1172 0.9%	17/5819 0.2%
7165	signal transduction	8.29E-05	1.27E-03	77/1172 6.5%	257/5819 4.4%
122	negative regulation of transcription from RNA polymerase II promoter	8.48E-05	1.29E-03	27/1172 2.3%	66/5819 1.1%
727	double-strand break repair via break-induced replication	8.74E-05	1.32E-03	7/1172 0.5%	8/5819 0.1%
43412	biopolymer modification	8.80E-05	1.32E-03	223/1172 19.0%	895/5819 15.3%
6357	regulation of transcription from RNA polymerase II promoter	1.36E-04	2.02E-03	71/1172 6.0%	236/5819 4.0%
7346	regulation of mitotic cell cycle	1.56E-04	2.29E-03	29/1172 2.4%	75/5819 1.2%
7534	gene conversion at mating-type locus	1.66E-04	2.42E-03	11/1172 0.9%	18/5819 0.3%
6810	transport	1.71E-04	2.48E-03	279/1172 23.8%	1162/5819 19.9%
43283	biopolymer metabolic process	1.77E-04	2.55E-03	567/1172 48.3%	2543/5819 43.7%
6473	protein amino acid acetylation	1.90E-04	2.70E-03	23/1172 1.9%	55/5819 0.9%
43044	ATP-dependent chromatin remodeling	2.41E-04	3.41E-03	15/1172 1.2%	30/5819 0.5%
43170	macromolecule metabolic process	2.56E-04	3.60E-03	676/1172 57.6%	3091/5819 53.1%
6289	nucleotide-excision repair	2.64E-04	3.67E-03	20/1172 1.7%	46/5819 0.7%
43285	biopolymer catabolic process	2.65E-04	3.67E-03	115/1172 9.8%	427/5819 7.3%
16359	mitotic sister chromatid segregation	2.72E-04	3.72E-03	25/1172 2.1%	63/5819 1.0%
278	mitotic cell cycle	2.73E-04	3.72E-03	84/1172 7.1%	295/5819 5.0%
31047	gene silencing by RNA	3.29E-04	4.45E-03	5/1172 0.4%	5/5819 0.0%
48468	cell development	3.42E-04	4.59E-03	17/1172 1.4%	37/5819 0.6%
6311	meiotic gene conversion	3.84E-04	5.12E-03	13/1172 1.1%	25/5819 0.4%
7533	mating type switching	3.89E-04	5.14E-03	14/1172 1.1%	28/5819 0.4%
8104	protein localization	4.44E-04	5.83E-03	135/1172 11.5%	520/5819 8.9%
6312	mitotic recombination	5.04E-04	6.57E-03	17/1172 1.4%	38/5819 0.6%
16571	histone methylation	5.13E-04	6.61E-03	10/1172 0.8%	17/5819 0.2%
33036	macromolecule localization	5.15E-04	6.61E-03	147/1172 12.5%	575/5819 9.8%
43543	protein amino acid acylation	5.40E-04	6.88E-03	26/1172 2.2%	69/5819 1.1%
9628	response to abiotic stimulus	5.69E-04	7.20E-03	45/1172 3.8%	141/5819 2.4%
43574	peroxisomal transport	5.86E-04	7.32E-03	11/1172 0.9%	20/5819 0.3%
6625	protein targeting to peroxisome	5.86E-04	7.32E-03	11/1172 0.9%	20/5819 0.3%
6354	RNA elongation	5.98E-04	7.42E-03	27/1172 2.3%	73/5819 1.2%
34262	macroautophagy	6.22E-04	7.66E-03	12/1172 1.0%	23/5819 0.3%
45184	establishment of protein localization	6.27E-04	7.67E-03	128/1172 10.9%	493/5819 8.4%
9058	biosynthetic process	6.38E-04	7.75E-03	411/1172 35.0%	1810/5819 31.1%
7033	vacuole organization and biogenesis	6.56E-04	7.92E-03	18/1172 1.5%	42/5819 0.7%
51649	establishment of localization in cell	6.95E-04	8.28E-03	158/1172 13.4%	628/5819 10.7%
6886	intracellular protein transport	6.96E-04	8.28E-03	84/1172 7.1%	303/5819 5.2%
15031	protein transport	7.00E-04	8.28E-03	125/1172 10.6%	481/5819 8.2%
44260	cellular macromolecule metabolic process	8.39E-04	9.87E-03	398/1172 33.9%	1753/5819 30.1%
6873	cellular ion homeostasis	8.59E-04	9.96E-03	38/1172 3.2%	116/5819 1.9%
55082	cellular chemical homeostasis	8.59E-04	9.96E-03	38/1172 3.2%	116/5819 1.9%
3006	reproductive developmental process	8.75E-04	9.96E-03	15/1172 1.2%	33/5819 0.5%
7531	mating type determination	8.75E-04	9.96E-03	15/1172 1.2%	33/5819 0.5%
7530	sex determination	8.75E-04	9.96E-03	15/1172 1.2%	33/5819 0.5%
7031	peroxisome organization and biogenesis	9.19E-04	1.04E-02	18/1172 1.5%	43/5819 0.7%
7059	chromosome segregation	9.38E-04	1.06E-02	45/1172 3.8%	144/5819 2.4%
51452	intracellular pH reduction	1.02E-03	1.12E-02	12/1172 1.0%	24/5819 0.4%
45851	pH reduction	1.02E-03	1.12E-02	12/1172 1.0%	24/5819 0.4%
7035	vacuolar acidification	1.02E-03	1.12E-02	12/1172 1.0%	24/5819 0.4%
16573	histone acetylation	1.03E-03	1.13E-02	17/1172 1.4%	40/5819 0.6%
30435	sporulation	1.10E-03	1.20E-02	48/1172 4.0%	157/5819 2.6%
19538	protein metabolic process	1.12E-03	1.21E-02	387/1172 33.0%	1706/5819 29.3%
75	cell cycle checkpoint	1.13E-03	1.21E-02	21/1172 1.7%	54/5819 0.9%
44267	cellular protein metabolic process	1.16E-03	1.25E-02	386/1172 32.9%	1702/5819 29.2%
87	M phase of mitotic cell cycle	1.25E-03	1.33E-02	51/1172 4.3%	170/5819 2.9%
6265	DNA topological change	1.27E-03	1.34E-02	6/1172 0.5%	8/5819 0.1%
19725	cellular homeostasis	1.28E-03	1.34E-02	48/1172 4.0%	158/5819 2.7%
30004	cellular monovalent inorganic cation homeostasis	1.28E-03	1.34E-02	15/1172 1.2%	34/5819 0.5%

6347	chromatin silencing at silent mating-type cassette	1.40E-03	1.46E-02	14/1172 1.1%	31/5819 0.5%
6334	nucleosome assembly	1.51E-03	1.57E-02	13/1172 1.1%	28/5819 0.4%
30641	regulation of cellular pH	1.60E-03	1.58E-02	12/1172 1.0%	25/5819 0.4%
51453	regulation of intracellular pH	1.60E-03	1.58E-02	12/1172 1.0%	25/5819 0.4%
16574	histone ubiquitination	1.64E-03	1.58E-02	4/1172 0.3%	4/5819 0.0%
45021	error-free DNA repair	1.64E-03	1.58E-02	4/1172 0.3%	4/5819 0.0%
32784	regulation of RNA elongation	1.64E-03	1.58E-02	4/1172 0.3%	4/5819 0.0%
32786	positive regulation of RNA elongation	1.64E-03	1.58E-02	4/1172 0.3%	4/5819 0.0%
433	negative regulation of transcription from RNA polymerase II promoter by glucose	1.64E-03	1.58E-02	5/1172 0.4%	6/5819 0.1%
437	RNA polymerase II promoter by carbon catabolites	1.64E-03	1.58E-02	5/1172 0.4%	6/5819 0.1%
429	polymerase II promoter by carbon catabolites	1.64E-03	1.58E-02	5/1172 0.4%	6/5819 0.1%
430	regulation of transcription from RNA polymerase II promoter by glucose	1.64E-03	1.58E-02	5/1172 0.4%	6/5819 0.1%
45013	negative regulation of transcription by carbon catabolites	1.64E-03	1.58E-02	5/1172 0.4%	6/5819 0.1%
45014	glucose	1.64E-03	1.58E-02	5/1172 0.4%	6/5819 0.1%
6358	regulation of transcription from RNA polymerase II promoter, global	1.64E-03	1.58E-02	5/1172 0.4%	6/5819 0.1%
45132	meiotic chromosome segregation	1.65E-03	1.58E-02	11/1172 0.9%	22/5819 0.3%
7067	mitosis	1.68E-03	1.60E-02	50/1172 4.2%	168/5819 2.8%
48610	reproductive cellular process	1.76E-03	1.67E-02	44/1172 3.7%	144/5819 2.4%
6310	DNA recombination	1.90E-03	1.79E-02	51/1172 4.3%	173/5819 2.9%
6139	nucleobase, nucleoside, nucleotide and nucleic acid metabolic process	2.02E-03	1.90E-02	400/1172 34.1%	1781/5819 30.6%
46907	intracellular transport	2.25E-03	2.10E-02	143/1172 12.2%	576/5819 9.8%
48518	positive regulation of biological process	2.33E-03	2.16E-02	46/1172 3.9%	154/5819 2.6%
6301	postreplication repair	2.48E-03	2.29E-02	8/1172 0.6%	14/5819 0.2%
30163	protein catabolic process	2.60E-03	2.39E-02	82/1172 6.9%	307/5819 5.2%
6260	DNA replication	2.65E-03	2.42E-02	47/1172 4.0%	159/5819 2.7%
16558	protein import into peroxisome matrix	2.68E-03	2.44E-02	9/1172 0.7%	17/5819 0.2%
51716	cellular response to stimulus	2.71E-03	2.45E-02	22/1172 1.8%	61/5819 1.0%
48878	chemical homeostasis	2.84E-03	2.55E-02	38/1172 3.2%	123/5819 2.1%
50801	ion homeostasis	2.84E-03	2.55E-02	38/1172 3.2%	123/5819 2.1%
44248	cellular catabolic process	2.87E-03	2.55E-02	146/1172 12.4%	593/5819 10.1%
6730	one-carbon compound metabolic process	2.88E-03	2.55E-02	27/1172 2.3%	80/5819 1.3%
22607	cellular component assembly	3.06E-03	2.71E-02	63/1172 5.3%	227/5819 3.9%
30491	heteroduplex formation	3.16E-03	2.75E-02	6/1172 0.5%	9/5819 0.1%
6283	repair	3.16E-03	2.75E-02	6/1172 0.5%	9/5819 0.1%
46173	polyol biosynthetic process	3.16E-03	2.75E-02	6/1172 0.5%	9/5819 0.1%
7093	mitotic cell cycle checkpoint	3.24E-03	2.81E-02	13/1172 1.1%	30/5819 0.5%
10564	regulation of cell cycle process	3.42E-03	2.95E-02	22/1172 1.8%	62/5819 1.0%
15992	proton transport	3.51E-03	3.02E-02	15/1172 1.2%	37/5819 0.6%
51242	positive regulation of cellular process	3.59E-03	3.07E-02	45/1172 3.8%	153/5819 2.6%
6348	chromatin silencing at telomere	3.73E-03	3.15E-02	20/1172 1.7%	55/5819 0.9%
31509	telomeric heterochromatin formation	3.73E-03	3.15E-02	20/1172 1.7%	55/5819 0.9%
65009	regulation of molecular function	3.76E-03	3.17E-02	30/1172 2.5%	93/5819 1.5%
9057	macromolecule catabolic process	3.81E-03	3.20E-02	121/1172 10.3%	484/5819 8.3%
44257	cellular protein catabolic process	3.97E-03	3.31E-02	78/1172 6.6%	294/5819 5.0%
32879	regulation of localization	4.03E-03	3.35E-02	7/1172 0.5%	12/5819 0.2%
9056	catabolic process	4.36E-03	3.61E-02	151/1172 12.8%	622/5819 10.6%
32787	monocarboxylic acid metabolic process	4.41E-03	3.63E-02	40/1172 3.4%	134/5819 2.3%
46015	regulation of transcription by glucose	4.80E-03	3.88E-02	5/1172 0.4%	7/5819 0.1%
45144	meiotic sister chromatid segregation	4.80E-03	3.88E-02	5/1172 0.4%	7/5819 0.1%
7135	meiosis II	4.80E-03	3.88E-02	5/1172 0.4%	7/5819 0.1%
50000	chromosome localization	4.80E-03	3.88E-02	5/1172 0.4%	7/5819 0.1%
16192	vesicle-mediated transport	4.93E-03	3.97E-02	90/1172 7.6%	349/5819 5.9%
7062	sister chromatid cohesion	5.48E-03	4.39E-02	14/1172 1.1%	35/5819 0.6%
19751	polyol metabolic process	5.77E-03	4.60E-02	11/1172 0.9%	25/5819 0.4%
45935	nucleoside, nucleotide and nucleic acid metabolic process	5.84E-03	4.64E-02	40/1172 3.4%	136/5819 2.3%
55067	monovalent inorganic cation homeostasis	6.30E-03	4.99E-02	15/1172 1.2%	39/5819 0.6%
