# Supplementary material for: Identification of Novel Human Damage Response Proteins Targeted through Yeast Orthology
Source: PLoS One. 2012 May 16;7(5):e37368. doi: 10.1371/journal.pone.0037368 (PMC3353887; doi:10.1371/journal.pone.0037368)
Supplement: Table S2 — Summary of the human potential toxicity-modulating proteins. The summary includes the described function in the cell and the yeast homologs, together with a toxicity-modulation summary of both yeast and human cells. (PDF) [file pone.0037368.s004.pdf]

Table S2

Symbol	Name	Summary Function (from NCBI Entrez)	Gene Ontology	homolog	yeast 4NQO	yeast MMS	yeast UV	yeast tOH	human 293T 4NQO	human 293T MMS	human 293T tOH
**ACD**	Adrenocortical dysplasia homolog (mouse)	involved in telomere function. ACD is one of six core proteins in the telosome/shelterin telomeric complex, which functions to maintain telomere length and to protect telomere ends. Through its interaction with other components, ACD plays a key role in the assembly and stabilization of this complex, and it mediates the access of telomerase to the telomere.	morphogenesis|intracellular protein transport|negative regulation of telomere maintenance via telomerase|positive regulation of single-stranded telomeric DNA binding|positive regulation of telomerase activity|protection from non-homologous end joining at telomere|segmentation|skeletal system development|telomere assembly|urogenital system development	no homolog					0	-1	2
**ADK**	Adenosine kinase	an abundant enzyme in mammalian tissues. ADK catalyzes the transfer of the gamma-phosphate from ATP to adenosine, thereby serving as a regulator of concentrations of extracellular adenosine and intracellular adenine. Adenosine has widespread effects on the cardiovascular, nervous, respiratory, and immune systems and inhibitors of the enzyme could play an pharmacological role in increasing intravascular adenosine concentrations and acting as anti-inflammatory agents.	adenosine kinase activity|kinase activity|magnesium ion binding|transferase activity|purine ribonucleoside salvage|ribonucleoside monophosphate biosynthetic process	YJR105W	23	2	0	0	0	0	0
**ASF1B**	ASF1 anti-silencing function 1 homolog B (S. cerevisiae)	a member of the H3/H4 family of histone chaperone proteins and is similar to the anti-silencing function-1 gene in yeast. The encoded protein is the substrate of the tousled-like kinase family of cell cycle-regulated kinases, and may play a key role in modulating the nucleosome structure of chromatin by ensuring a constant supply of histones at sites of nucleosome assembly.	histone binding|cell differentiation|chromatin assembly or disassembly|chromatin modification|multicellular organismal development|nucleosome assembly|regulation of transcription, DNA-dependent|spermatogenesis|transcription|chromatin|nucleus	YJL115W	26	30	0	0	0	0	0
**ATP6V1D**	ATPase, H+ transporting, lysosomal 34kDa, V1 subunit D	component of vacuolar ATPase (V-ATPase), a multisubunit enzyme that mediates acidification of eukaryotic intracellular organelles. V-ATPase dependent organelle acidification is necessary for such intracellular processes as protein sorting, zymogen activation, receptor-mediated endocytosis, and synaptic vesicle proton gradient generation. V-ATPase is composed of a cytosolic V1 domain and a transmembrane V0 domain. The V1 domain consists of three A and three B subunits, two G subunits plus the C, D, E, F, and H subunits. The V1 domain contains the ATP catalytic site. The V0 domain consists of five different subunits: a, c, c', c", and d. Additional isoforms of many of the V1 and V0 subunit proteins are encoded by multiple genes or alternatively spliced transcript variants.		YEL051W	30	18	0	9	0	-2	-1
**ATP6V1F**	ATPase, H+ transporting, lysosomal 14kDa, V1 subunit F		ATPase activity, coupled to transmembrane movement of substances|proton-transporting ATPase activity, rotational mechanism|ATP synthesis coupled proton transport|ion transport|proton transport|proton-transporting two-sector ATPase complex, catalytic domain	YGR020C	22	7	6	2	0	1	1
**BCAP29**	B-cell receptor-associated protein 29		apoptosis|intracellular protein transport|vesicle-mediated transport|endoplasmic reticulum|endoplasmic reticulum membrane|integral to membrane|membrane	YDL072C	22	15	13	2	-		
**C14orf1**	open reading frame 1		sterol biosynthetic process|endoplasmic reticulum membrane|integral to membrane|transport vesicle	YER044C	26	30	9	16	0	0	2
**CDC40**	Cell division cycle 40 homolog	essential for the catalytic step II in pre-mRNA splicing process. In the spliceosome, and contains seven WD repeats, which function in protein-protein interactions. Sequence similarity to yeast Prp17p, which functions in pre-mRNA splicing and cell cycle progression.	RNA splicing|nuclear mRNA splicing, via spliceosome|nucleus|spliceosome	YDR364C	20	20	9	0	2	0	0
**CLTC**	Clathrin, heavy chain (Hc)	major protein component of the cytoplasmic face of intracellular organelles, called coated vesicles and coated pits. These specialized organelles are involved in the intracellular trafficking of receptors and endocytosis of a variety of macromolecules. The basic subunit of the clathrin coat is composed of three heavy chains and three light chains.	protein binding|structural molecule activity|intracellular protein transport|vesicle-mediated transport|clathrin coat of coated pit|clathrin coat of trans-Golgi network vesicle|cytoplasmic vesicle|cytoplasmic vesicle membrane|melanosome|plasma membrane	YGL206C	30	19	0	6	2	0	2
**CTDNEP1**	CTD nuclear envelope phosphatase 1		hydrolase activity|protein serine/threonine phosphatase activity|nuclear envelope organization|protein amino acid dephosphorylation|endoplasmic reticulum membrane|integral to membrane|nuclear membrane|	YHR004C	10	10	7	2	0	-2	-3
**EXOSC10**	Exosome component 10		[illegible] protein binding|protein serine/threonine kinase activity|nuclear-transcribed mRNA catabolic process, nonsense-mediated decay|nuclear exosome (RNase complex)	YOR001W	0	7	13	0	0	0	0
**FBXL2**	F-box and leucine-rich repeat protein 2	member of the F-box protein family, characterized by an approximately 40 amino acid motif, the F-box. F-box proteins constitute 1 of 4 subunits of ubiquitin protein ligase complex SCFs (SKP1-cullin-F-box), which function in phosphorylation-dep. ubiquitination. FBXL2, in addition to an F-box, contains 12 tandem leucine-rich repeats.	protein binding|ubiquitin-protein ligase activity|modification-dependent protein catabolic process|protein modification process|proteolysis|cytoplasm	YJR090C	6	5	3	5	0	-1	0
**HDAC6**	Histone deacetylase 6	belongs to class II of the histone deacetylase/acuc/apha family. It contains an internal duplication of two catalytic domains which appear to function independently of each other. This protein possesses histone deacetylase activity and represses transcription.	actin binding|hydrolase activity|metal ion binding|specific transcriptional repressor activity|tubulin deacetylase activity|zinc ion binding|cell cycle|chromatin modification|multicellular organismal development|negative regulation of microtubule depolymerization|protein polyubiquitination| regulation of transcription, DNA-dependent	YNL021W	12	5	0	2	0	0	0
**HSPBP1**	Hsp70-inter-acting protein		enzyme inhibitor activity|protein binding|protein folding	YBR101C	14	16	24	8	2	0	3
**LSM1**	LSM1 homolog, U6 small nuclear RNA associated (S. cerevisiae)	Sm-like proteins were identified in a variety of organisms based on sequence homology with the Sm protein family. Sm-like proteins contain the Sm sequence motif, which consists of 2 regions separated by a linker of variable length that folds as a loop. The Sm-like proteins are thought to form a stable heteromer present in tri-snRNP particles, which are important for pre-mRNA splicing.	RNA binding|RNA splicing factor activity, transesterification mechanism|protein binding|RNA splicing|mRNA processing|cytoplasm|nucleus|ribonucleoprotein complex	YJL124C	30	18	0	0	0	-1	0
**MED31**	Mediator complex subunit 31		RNA polymerase II transcription mediator activity|protein binding|regulation of transcription, DNA-dependent|mediator complex|nucleus	YGL127C	7	7	0	0	-		
**NCBP2**	Nuclear cap binding protein subunit 2, 20kDa	component of the nuclear cap-binding protein complex (CBC), which binds to the monomethylated 5' cap of nascent pre-mRNA in the nucleoplasm. NCBP2 has an RNP domain commonly found in RNA binding proteins, and contains the cap-binding activity. The CBC promotes pre-mRNA splicing, 3'-end processing, RNA nuclear export, and nonsense-mediated mRNA decay.	RNA cap binding|nucleotide binding|protein binding|RNA splicing|mRNA transport|positive regulation of RNA export from nucleus|snRNA export from nucleus|snRNA export from nucleus|spliceosomal snRNP biogenesis| transport|cytosol|mRNA cap binding complex|nucleoplasm|nucleus|nucleus	YPL178W	12	7	0	0	0	0	0
**PEX10**	Peroxisome biogenesis factor 10	involved in import of peroxisomal matrix proteins. This protein localizes to the peroxisomal membrane. Mutations in this gene result in phenotypes within the Zellweger spectrum of peroxisomal biogenesis disorders, ranging from neonatal adrenoleukodystrophy to Zellweger syndrome.	metal ion binding|protein binding|zinc ion binding|peroxisome organization|protein import into peroxisome matrix|membrane|peroxisomal membrane|peroxisome	YDR265W	0	12	0	0	0	0	2
**PFDN5**	Prefoldin subunit 5	member of the prefoldin alpha subunit family. PFDN5 is one of six subunits of prefoldin, a molecular chaperone complex that binds and stabilizes newly synthesized polypeptides, allowing them to fold correctly. The complex, consisting of two alpha and four beta subunits, forms a double beta barrel assembly with six protruding coiled-coils. The encoded protein may also repress the transcriptional activity of the proto-oncogene c-Myc.	transcription corepressor activity|unfolded protein binding|protein folding|regulation of transcription, DNA-dependent|cytoplasm|cytoskeleton|nucleus|prefoldin complex	YML094W	26	18	0	0	-1	-1	0
**POLR2D**	Polymerase (RNA) II (DNA directed) polypeptide D	fourth largest subunit of RNA polymerase II, the polymerase responsible for synthesizing messenger RNA in eukaryotes. In yeast, this polymerase subunit is associated with the polymerase under suboptimal growth conditions and may have a stress protective role. A sequence for a ribosomal pseudogene is contained within the 3' untranslated region of the transcript from this gene.	DNA-directed RNA polymerase activity|catalytic activity|nucleotide binding|RNA elongation from RNA polymerase II promoter|cellular metabolic process|nuclear mRNA splicing, via spliceosome|transcription|transcription initiation from RNA polymerase II promoter|DNA-directed RNA polymerase II, core complex|nucleoplasm|nucleus	YJL140W	23	22	8	4	2	-1	2
**POT1**	POT1 protection of telomeres 1 homolog (S. pombe)	a member of the telombin family and is involved in telomere maintenance. POT1 functions as a member of a multi-protein complex that binds to the TTAGGG repeats of telomeres, regulating telomere length and protecting chromosome ends from recombination, chromosome instability, and abnormal chromosome segregation. Increased transcriptional expression associated with stomach carcinogenesis and its progression.	DEAD/H-box RNA helicase binding|DNA binding|protein binding|single-stranded telomeric DNA binding|telomerase inhibitor activity|DNA duplex unwindingnegative regulation of telomere maintenance via telomerase|positive regulation of DNA strand elongation|positive regulation of helicase activity|positive regulation of telomerase activity	no homolog					0	-1	1
**PRPS2**	Phosphoribosyl pyrophosphate synthetase 2		kinase activity|magnesium ion binding|ribose phosphate diphosphokinase activity|transferase activity|cellular biosynthetic process|nucleobase, nucleoside, nucleotide and nucleic acid metabolic process|nucleoside metabolic process|nucleotide biosynthetic process|ribonucleoside monophosphate biosynthetic process	YHL011C	10	4	8	3	0	0	0
**RNASEH2A**	Ribonuclease H2, subunit A	Of the multiple RNases H in mammals, RNase HI is the major enzyme and shows increased activity during DNA replication. It shows more homology to the RNase HII of Escherichia coli.	RNA binding|endonuclease activity|hydrolase activity|metal ion binding|ribonuclease H activity|ribonuclease activity|DNA replication|RNA catabolic process|nucleus	YNL072W	8	5	0	0	0	1	1
**RNF20**	Ring finger protein 20	similarity with BRE1 of S. cerevisiae. Yeast BRE1 is a ubiquitin ligase required for the ubiquitination of histone H2B and the methylation of histone H3.	ligase activity|protein binding|zinc ion binding|chromatin modification|modification-dependent protein catabolic process	YDL074C	30	15	0	0	2	0	2
**SACM1L**	SAC1 suppressor of actin mutations 1-like		hydrolase activity|phosphatase activity|biological_process|Golgi apparatus|endoplasmic reticulum|endoplasmic reticulum membrane|integral to membrane|membrane	YKL212W	30	4	2	7	2	2	3
**SC5DL**	Sterol-C5-desaturase-like	enzyme of cholesterol biosynthesis. SC5DL catalyzes the conversion of lathosterol into 7-dehydrocholesterol. Mutations have been associated with lathosterolosis.	C-5 sterol desaturase activity|iron ion binding|lathosterol oxidase activity| oxidoreductase activity|fatty acid biosynthetic process|oxidation reduction|sterol biosynthetic process|endoplasmic reticulum|endoplasmic reticulum membrane	YLR056W	24	11	6	8	0	0	2

**SIN3A**	A, transcription regulator (yeast)	transcriptional regulatory protein. It contains paired amphipathic helix (PAH) domains, which are important for protein-protein interactions and may mediate repression by the Mad-Max complex.	DNA binding|transcription corepressor activity|transcription factor binding|negative regulation of transcription from RNA polymerase II promoter|transcription|kinetochore|nucleus	YOL004W	26	10	0	0	2	0	2
**SNF8**	SNF8, ESCRT-II complex subunit, homolog	SNF8, VPS25, and VPS36 form ESCRT-II (endosomal sorting complex required for transport II), a complex involved in endocytosis of ubiquitinated membrane proteins. SNF8, VPS25, and VPS36 are also associated in a multiprotein complex with RNA polymerase II elongation factor.	RNA polymerase II transcription factor activity|protein transport|regulation of transcription from RNA polymerase II promoter|transcription|cytoplasm|nucleus	YPL002C	0	26	0	2	0	0	0
**STX12**	Syntaxin 12		SNAP receptor activity|cholesterol efflux|intracellular protein transport|protein stabilization|vesicle-mediated transport|Golgi apparatus|integral to membrane|membrane|membrane raft|phagocytic vesicle	YOR036W	0	5	5	2	1	0	1
**TADA2L**	Transcriptional adaptor 2-like	transcriptional activator adaptor and has been found to be part of the PCAF histone acetylase complex. Many activator proteins enhance the initiation rate of RNA polymerase II-mediated transcription by interacting with general transcription machinery bound at promoter. Adaptor proteins are usually required to acetylate and destabilize nucleosomes, thereby relieving chromatin constraints at the promoter.	transcription cofactor activity|transcription factor activity|zinc ion binding|regulation of transcription, DNA-dependent|transcription from RNA polymerase II promoter|chromosome|nucleus	YDR448W	0	4	6	0	-2	1	0
**TBL1XR1**	Transducin (beta)-like 1 X-linked receptor 1	sequence similarity with members of the WD40 repeat-containing protein family. The WD40 group is a large family of proteins with regulatory function. WD40 repeats mediate protein-protein interactions and members of the family are involved in signal transduction, RNA processing, gene regulation, vesicular trafficking, cytoskeletal assembly and possibly control of cytotypic	chromatin modification|modification-dependent protein catabolic process|regulation of transcription, DNA-dependent|transcription|nucleus	YBR103W	0	12	0	2	0	1	0
**TERF1**	Telomeric repeat binding factor (NIMA-interacting) 1	telomere specific protein which is a component of the telomere nucleoprotein complex. This protein is present at telomeres throughout the cell cycle and functions as an inhibitor of telomerase, acting in cis to limit the elongation of individual chromosome ends. The protein structure contains a C-terminal Myb motif, a dimerization domain near its N-terminus and an acidic N-terminus.	DNA bending activity|double-stranded telomeric DNA binding|protein homodimerization activity|telomerase inhibitor activity|cell cycle|cell division|mitosis|negative regulation of telomere maintenance via semi-conservative replication|negative regulation of telomere maintenance via telomerase|regulation of transcription|telomere maintenance via telomere shortening|spindle	no homolog					0	-1	1
**TERF2**	Telomeric repeat binding factor 2	telomere specific protein, TERF2, which is a component of the telomere nucleoprotein complex. This protein is present at telomeres in metaphase of the cell cycle, is a second negative regulator of telomere length and plays a key role in the protective activity of telomeres. While having similar telomere binding activity and domain organization, TERF2 differs from TERF1 in that its N terminus is basic rather than acidic.	double-stranded telomeric DNA binding|age-dependent telomere shortening|cell cycle|in utero embryonic development|negative regulation of telomere maintenance via semi-conservative replication|positive regulation of telomere maintenance|protection from non-homologous end joining at telomere|regulation of transcription| senescence|telomere maintenance via telomerase|telomeric loop formation|Golgi apparatus|Mre11 complex|male germ cell nucleus	no homolog					-1	-2	2
**TMLHE**	Trimethyllysine hydroxylase, epsilon		L-ascorbic acid binding|electron carrier activity|iron ion binding|oxidoreductase activity|oxidoreductase activity, acting on single donors with incorporation of molecular oxygen, incorporation of two atoms of oxygen|trimethyllysine dioxygenase activity|carnitine biosynthetic process|oxidation reduction|mitochondrial matrix|mitochondrial matrix|mitochondrion	YHL021C	7	7	3	2	2	0	2
**TOP3A**	Topoisomerase (DNA) III alpha	a DNA topoisomerase, an enzyme that controls and alters the topologic states of DNA during transcription. TOP3A catalyzes the transient breaking and rejoining of a single strand of DNA which allows the strands to pass through one another, thus reducing the number of supercoils and altering the topology of DNA. This enzyme forms a complex with BLM which functions in the regulation of recombination in somatic cells.	DNA topoisomerase type I activity|metal ion binding|protein binding|zinc ion binding|DNA topological change|DNA unwinding during replication|meiosis|PML body|chromosome|nucleus	YLR234W	30	30	9	0	2	0	2
**TSG101**	Tumor susceptibility gene 101	belongs to a group of apparently inactive homologs of ubiquitin-conjugating enzymes. TSG101 contains a coiled-coil domain that interacts with stathmin, a cytosolic phosphoprotein implicated in tumorigenesis. TSG101 may play a role in cell growth and differentiation and act as a negative growth regulator. In vitro steady-state expression of this tumor susceptibility gene appears to be important for maintenance of genomic stability and cell cycle regulation. Mutations and alternative splicing in this gene occur in high frequency in breast cancer and suggest that defects occur during breast cancer tumorigenesis and/or progression.	DNA binding|protein binding|small conjugating protein ligase activity|transcription corepressor activity|ubiquitin binding|cell cycle arrest|interspecies interaction between organisms|keratinocyte differentiation|negative regulation of cell proliferation|negative regulation of transcription, DNA-dependent|non-lytic virus budding|post-translational protein modification|protein transport|regulation of cell growth|regulation of protein metabolic process|ubiquitin-dependent protein catabolic process via the multivesicular body sorting pathway|cytoplasm|late endosome membrane|multivesicular body|nucleolus|nucleus|plasma	YCL008C	0	22	0	7	0	0	0
**TTF2**	Transcription termination factor, RNA polymerase II	a member of the SWI2/SNF2 family of proteins, which play a critical role in altering protein-DNA interactions. TTF2 has dsDNA-dependent ATPase activity and RNA polymerase II termination activity. TTF2 interacts with cell division cycle 5-like, associates with human splicing complexes, and plays a role in pre-mRNA splicing.	ATP-dependent helicase activity|DNA-dependent ATPase activity|RNA polymerase II transcription termination factor|hydrolase activity|nucleotide binding|zinc ion binding|RNA splicing|transcription|transcription elongation factor complex	YLR032W	30	30	25	0	0	0	0
**VPS16A**	Vacuolar protein sorting 16 homolog A	vesicle mediated protein sorting plays an important role in segregation of intracellular molecules into distinct organelles. The mammalian class C Vps proteins are predominantly associated with late endosomes/lysosomes and may mediate vesicle trafficking steps in the endosome/lysosome pathway.	intracellular protein transport, endosome, late endosome, lysosomal membrane	YPL045W	21	15	0	13	0	-2	0
**XPA**	Xeroderma pigmentosum, complementation group A	a zinc finger protein involved in DNA excision repair. XPA is part of the NER (nucleotide excision repair) complext which is responsible for repair of UV radiation-induced photoproducts and DNA adducts induced by chemical carcinogens. Mutations in this gene are associated with xeroderma pigmentosum complementation group A.	damaged DNA binding|metal ion binding|nucleotide binding|protein domain specific binding|protein homodimerization activity|zinc ion binding|nucleotide-excision repair, DNA damage removal|response to DNA damage stimulus|nucleoplasm|nucleus	YMR201C	29	7	22	0	-3	0	0
**ZFYVE20**	Zinc finger, FYVE domain containing 20		early endosome membrane, endosome transport, protein transport, plasma membrane	YDR323C	26	10	4	2	3	2	3
**SLC25A5**	Solute carrier family 25 (mito-chondrial carrier; adenine trans-locator)membe r5	ADP/ATP translocase, the most abundant mitochondrial protein, is an integral component of the inner mitochondrial membrane. It facilitates exchange of ADP and ATP between the cytosol and the mitochondria, thereby linking the subcellular compartment of ATP production to those of ATP utilization. SLC25A5 is 1 of at least 3 transcriptionally active ADP/ATP translocase genes in humans.	adenine transmembrane transporter activity|binding|transporter activity|interspecies interaction between organisms|transport|integral to plasma membrane|membrane|mitochondrial inner membrane|mitochondrial inner membrane|mitochondrial nucleoid|mitochondrion	YBR085W	0	0	0	0			
**AP3D1**	Adaptor-related protein complex 3, delta 1 subunit	AP3D1 is a subunit of the AP3 adaptor-like complex, which is not associated with clathrin. The AP3D1 subunit is implicated in intracellular biogenesis and trafficking of pigment granules and possibly platelet dense granules and neurotransmitter vesicles.	protein binding|protein transporter activity|transporter activity|eye pigment biosynthetic process|intracellular protein transport|vesicle-mediated transport|Golgi apparatus|Golgi membrane|membrane|membrane coat	YPL195W	0	0	0	0			
**ADIPOR1**	Adiponectin receptor 1	ADIPOR1 and ADIPOR2 serve as receptors for globular and full length adiponectin and mediate increased AMPK and PPAR-alpha ligand activities, as well as fatty acid oxidation and glucose uptake by adiponectin.	hormone binding|receptor activity|fatty acid oxidation|hormone-mediated signaling|lipid metabolic process|integral to membrane|membrane	YDR492W	0	0	0	0			
**URM1**	related modifier 1 homolog		modification-dependent protein catabolic process	YIL008W	0	0	0	0			
**RASA1**	RAS p21 protein activator 1	part of the GAP1 family of GTPase-activating proteins. RASA1 stimulates the GTPase activity of normal RAS p21 but not its oncogenic counterpart. This suppressor of RAS function enhances the weak GTPase activity of RAS proteins resulting in the inactive GDP-bound form of RAS, thereby allowing control of cellular proliferation and differentiation. Mutations leading to changes in the binding sites are associated with basal cell carcinomas.	binding|cytokinesis|embryonic development|intracellular signaling cascade|negative regulation of cell adhesion|negative regulation of cell-matrix adhesion|negative regulation of neuron apoptosis|regulation of RNA metabolic process|regulation of actin filament polymerization|regulation of cell shape|regulation of small GTPase mediated signal transduction|vasculogenesis|plasma membrane|ruffle	YKL092C	0	0	0	0			

Yeast sensitivity scores from Begley et al, Mol. Cell. 2004

20-30	high sensitivity	1-3	resistant
2-19	low-medium sensitivity	-3- -1	sensitive
