# Supplementary material for: Identification of Novel Human Damage Response Proteins Targeted through Yeast Orthology
Source: PLoS One. 2012 May 16;7(5):e37368. doi: 10.1371/journal.pone.0037368 (PMC3353887; doi:10.1371/journal.pone.0037368)
Supplement: Table S3 — shRNA constructs and qRT-PCR primers. (PDF) [file pone.0037368.s005.pdf]

Table S3

symbol	Identifier	Entrez Gene Id	Open Biosystems catalog number	oligo ID	PCR primer sequence left	PCR primer sequence left	code (293T)	293T KD efficiency	code (TK6)	TK6 KD efficiency
ACD	NM_022914	65057	RHS4430-98524277	V2LHS_116356	ccctgctctgtctgggaac	atgagctggggaggaagc	42.1	0.0816	42.2	0.7169
ADIPOR1	NM_00112768	51094	RHS4430-98714358	V2LHS_134726	cgacgaacgtcctctaccc	gtccagtttggtcttctaaactcc	47.1	0.44288		
ADK	NM_006721	132	RHS4430-99329088	V2LHS_1191	gctgcatgcatcactggt	tctccagatcaagatgtttttcc	19.3	0.25525		
AP3D1	NM_001077523	8943	RHS4430-98485767	V2LHS_47150	gctgaccaccaatcagatcc	agacccgtcagtgcaacac	46.2	1		
ASF1B	NM_018154	55723	RHS4430-99142050	V2LHS_156005	accctgagctgcgtgaga	tcgaggccaagatgttcc	28.1	0.1875	28.6	0.2449
ATP6V1D	NM_015994	51382	RHS4430-98852136	V2LHS_134703	ttttcactagctgaagccaagtt	gcgctttattgacattttggat	4.7	0.3597	4.1	0.19
ATP6V1D	NM_015994	51382	RHS4430-98853811	V2LHS_134698	ttttcactagctgaagccaagtt	gcgctttattgacattttggat	51.3	0.1678		
ATP6V1F	NM_004231	9296	RHS4430-98843664	V2LHS_28381	gaaccgccatcccaattt	caatgtcatcccggtttagaa	7.7	0.01	7.7	0.32
BCAP29	NM_018844	55973	RHS4430-98852036	V2LHS_21873	ctgcctaccttttattcctcctc	ttgcaattttaccccagaca	23	0.65067	23.5	0.4601
C14ORF1	NM_007176	11161	RHS4430-98521091	V2LHS_207531	tgccattgacattcacaaca	gacaaacaactcagagaggaaatg	36.1	0.46652		
CDC40	NM_015891	51362	RHS4430-98853717	V2LHS_227739	tctctagcagtggcagtgga	gggtcaaggtgaactccagtc	11.2	0.01105		
CLTC	NM_004859	1213	RHS4430-98705117	V2LHS_67882	tgggttgatacccaggttct	ccagattctgccaattcgtt	5.7	0.0431		
DULLARD	NM_015343	23399	RHS4430-98820118	V2LHS_74456	gatggggatggcattgtct	actttggagttgggcagcta	31.2	0.0205	31.2	0.695
DULLARD	NM_015343	23399	RHS4430-99329001	V2LHS_74457	gatggggatggcattgtct	actttggagttgggcagcta	-	-		
EXOSC10	NM_001001998	5394	RHS4430-98714946	V2LHS_170175	gaagttcgatccatcaaccaa	ttgacagcttctttagagtctttttg	18.6	0.1843	18.3	0.2466
FBXL2	NM_012157	25827	RHS4430-98910869	V2LHS_202584	gttacccgtgcaggcatc	aagtaggcgtggactttgaca	30.2	0.333		
HDAC6	NM_006044	10013	RHS4430-98912627	V2LHS_71189	agttcaccttcgaccaggac	gccagaacctaccctgctc	35.3	1		
HSPBP1	NM_012267	23640	RHS4430-98526302	V2LHS_240240	ccctcttcgccatctcct	aacacagagaagccgtccag	38.1	0.05256		
LSM1	NM_014462	27257	RHS4430-98851667	V2LHS_197725	ttcctcgagggatttttgtg	ctggaggggtgtgtcactct	15.2	0.2382	15.4	0.0951
MED31	NM_016060	40577	RHS4430-98819243	V2LHS_97357	aaagacccagaatatgccaagt	aatttcgcacactgagcattc	22	0.97		
NCBP2	NM_007362	22916	RHS4430-99139143	V2LHS_85841	ttaaggagggcaggcaatac	tctgtgccagttttccatagc	14.7	0.3523		
PEX10	NM_002617	5192	RHS4430-99160793	V2LHS_169849	ctgctctcagatgtggccta	ccacctggatgatgctgac	21.1	0.1146		
PFDN5	NM_002624	5204	RHS4430-98715886	V2LHS_169886	cagatggagaaaatccaaccag	ttctgactcatcatttccatgac	20.3	0.09539		
POLR2D	NM_004805	5433	RHS4430-99138856	V2LHS_68896	tccaaggctctaatcccaag	ttcataagtttgagttggcctgt	27.1	0.426317		0.1330/1
POLR2D	NM_004805	5433	RHS4430-98895913	V2LHS_68893	tccaaggctctaatcccaag	ttcataagtttgagttggcctgt	53.2	0.4414		
POT1	NM_015450	25913	RHS4430-98853211	V2LHS_96575	aatggagatattgttcgctttca	tggtgataccctgagtctcctt	43.1	0.3829		
PRPS2	NM_002765	5634	RHS4430-98820785	V2LHS_170588	ggtcacgaagaagttcagca	atcttcccctctcacgcttt	17.3	0.13167	17.3	0.5529
RASA1	NM_002890	5921	RHS4430-98704453	V2LHS_149857	gaggaagaggtggccatacc	tctgtcaagttttccgtgatacc	49.1	0.368509		
RNASEH2A	NM_006397	10535	RHS4430-98901475	V2LHS_196963	cccagtttgtccggttca	gattctcggatgctgagtcc	26.1	0.6329	26.2	0.7071
RNF20	NM_019592	56254	RHS4430-98486410	V2LHS_52294	aggggtgagagctggaatct	ctccagctgctcttttatttcc	16.5	0.2059	16.6	0.34032
SACM1L	NM_014016	22908	RHS4430-98708493	V2LHS_60317	aagtgttccaagggactgga	tgaaaaggcaacaaccatga	29.9	0.3242		
SC5DL	NM_006918	6309	RHS4430-98484748	V2LHS_94843	gacagtttgcagagcagtgg	cacggagtacaagatccatcac	37.1	0.3253	37.1	0.5415
SIN3A	NM_015477	25942	RHS4430-99160346	V2LHS_96678	acagaagaggagaattcggatg	cgctccacgtagtctgacc	10.2	0.1534	10.2	0.7684
SLC25A5	NM_001152	292	RHS4430-98894996	V2LHS_94497	ggcaacagcagtgacagtct	ctaccgagccgcctacttc	45.2	0.02855		
SNF8	NM_007241	11267	RHS4430-98852714	V2LHS_197270	tgagctcaatatggatcacacc	actgacagtcacgtagccattc	12.2	0.1476		
STX12	NM_177424	23673	RHS4430-98851408	V2LHS_207565	tctgcagaagagaggcaaaga	gttccactcctcatggctgt	8.8	0.3427	8.6	0.49
TADA2L	NM_133439	6871	RHS4430-98817870	V2LHS_131912	gcagaggaagcaaaaactgc	ggggagggtcatctgtagagt	9.1	0.08657	9.1	0.3882
TADA2L	NM_133439	6871	RHS4430-98902137	V2LHS_131910	gcagaggaagcaaaaactgc	ggggagggtcatctgtagagt	50.2	0.3737		
TBL1XR1	NM_024665	79718	RHS4430-98526016	V2LHS_157133	ctggaatgcagcaggagac	gcgctatttccgaaggtcta	33.3	1	33.2	0.895
TERF1	NM_003218	7013	RHS4430-99619369	V2LHS_153893	ctaacagcttgccagttgagaa	tcattttcaaactgtgcatcaa	40.3	0.2535		
TERF2	NM_005652	7014	RHS4430-99151616	V2LHS_202445	cccaccgttctcaaccaa	gttccacttgcctttgggta	41.1	0.1441	40.6	0.76313
TMLHE	NM_018196	55217	RHS4430-98894566	V2LHS_156237	tctgcggagttagcagtcac	ctgctgtgtaggtgggacaa	13.3	0.5783		
TOP3A	NM_004618	7156	RHS4430-98895260	V2LHS_42272	tttctggacatttactggctca	tgcttcaaagaggacaagagg	24.2	0.2755		
TSG101	NM_006292	7251	RHS4430-99290818	V2LHS_56429	tcctatttcggcatcctatcc	tgcctggcatgtaggaagta	34.2	0.895	34.3	0.7422
TTF2	NM_003594	8458	RHS4430-99165226	V2LHS_32324	agacggttgtggcagaagat	caatgcctgcttctggtgta	25.1	0.1199	25.5	0.6552
URM1	NM_030914	81605	RHS4430-98841140	V2LHS_137618	tgtttcttaataccgtcaaacagg	agctcggcttcctcaacat	48.4	0.1901		
VPS16	NM_022575	64601	RHS4430-98480960	V2LHS_74243	ggtcagctgaggaggagcta	ccggttctggagcacttc	6.7	0.1743	6.2	0.5864
XPA	NM_000380	7507	RHS4430-99330071	V2LHS_92696	cgagtatcgagcggaagc	tttggggctgcttttacatt	44.2	0.0269	44.4	0.2882
ZFYVE20	NM_022340	64145	RHS4430-99292164	V2LHS_87877	ggagccatggacactgttg	agccttcccttggcaaac	32.2	0.2124	32	1
ZFYVE20	NM_022341	64145	RHS4430-98520395	V2LHS_87876	ggagccatggacactgttg	agccttcccttggcaaac	54.3	0.2199		
Betaactin	NM_001101				ccaaccgcgagaagatga	ccagaggcgtacagggatag				
